# Temporal Expression Profiles Reveal Potential Targets during Postembryonic Development of Forensically Important *Sarcophaga peregrina* (Diptera: Sarcophagidae)

**DOI:** 10.3390/insects13050453

**Published:** 2022-05-12

**Authors:** Lipin Ren, Yanjie Shang, Xiangyan Zhang, Shan Chen, Yunna Zheng, Ying Zou, Yihong Qu, Jifeng Cai, Changquan Zhang, Yadong Guo

**Affiliations:** 1 Department of Forensic Science, School of Basic Medical Sciences, Central South University, Changsha 410013, China; renlp87@126.com (L.R.); shangyj@csu.edu.cn (Y.S.); zxy196@csu.edu.cn (X.Z.); zouhawk@163.com (Y.Z.); quyihong88@163.com (Y.Q.); cjf_jifeng@163.com (J.C.); 2 School of Ecological and Environmental Sciences, East China Normal University, Shanghai 200000, China; 52183903004@stu.ecnu.edu.cn; 3 OE Biotech Co., Ltd., Shanghai 200120, China; yunna.zheng@oebiotech.com

**Keywords:** *Sarcophaga peregrina*, metamorphosis, gene expression, age estimation

## Abstract

**Simple Summary:**

Forensic entomology plays a major role in postmortem interval (PMI) estimation, mainly based on morphological characteristics of larvae colonized on decomposed corpses. However, weight and length cannot be used as relevant parameters to calculate the age of wandering at the larval and pupal stages. In order to identify a specific indicator that may serve as a potential target, studies on the mechanism during postembryonic development may provide a foundation to establish a molecular age determination method. *Sarcophaga peregrina* (Robineau-Desvoidy, 1830) is a species of medical and forensic importance. In this study, transcriptome analysis was performed to investigate gene expression profiles from the larval to pupal stages of *S. peregrina*, which were used to identify differentially expressed genetic markers as candidates for molecular age estimation. This study explores molecular mechanisms during postembryonic development of *S. peregrina*. The developmental, stage-specific gene profiles suggest genetic markers for age prediction of forensically important flies.

**Abstract:**

*Sarcophaga peregrina* (Robineau-Desvoidy, 1830) is a species of medical and forensic importance. In order to investigate the molecular mechanism during postembryonic development and identify specific genes that may serve as potential targets, transcriptome analysis was used to investigate its gene expression dynamics from the larval to pupal stages, based on our previous *de novo*-assembled genome of *S. peregrina*. Totals of 2457, 3656, 3764, and 2554 differentially expressed genes were identified. The specific genes encoding the structural constituent of cuticle were significantly differentially expressed, suggesting that degradation and synthesis of cuticle-related proteins might actively occur during metamorphosis. Molting (20-hydroxyecdysone, 20E) and juvenile (JH) hormone pathways were significantly enriched, and gene expression levels changed in a dynamic pattern during the developmental stages. In addition, the genes in the oxidative phosphorylation pathway were significantly expressed at a high level during the larval stage, and down-regulated from the wandering to pupal stages. Weighted gene co-expression correlation network analysis (WGCNA) further demonstrated the potential regulation mechanism of tyrosine metabolism in the process of puparium tanning. Moreover, 10 consistently up-regulated genes were further validated by qRT-PCR. The utility of the models was then examined in a blind study, indicating the ability to predict larval development. The developmental, stage-specific gene profiles suggest novel molecular markers for age prediction of forensically important flies.

## 1. Introduction

Forensic entomology plays a major role in the investigation of decomposed corpses; the age determination of necrophagous flies is one of the key tasks in providing evidence for postmortem interval (PMI) estimation [1]. Generally, the developmental patterns of immature insects take place in a predictable manner, under a controlled temperature. So far, the age estimation of larvae has mostly relied on morphological parameters, such as the number of clefts in the posterior spiracle, as well as the length and weight (i.e., body size), which are subsequently compared with the species-specific reference growth data obtained from laboratory studies [2,3]. However, once the third instar larvae enter the wandering stage, they begin to shrink and exhibit increased variance in body size, making it difficult to use body size for age estimation [3,4]. Likewise, it is difficult to calculate the size measurements of pupation, which can last up to half of the total juvenile developmental phase, without external morphological changes [3]. Additionally, temperature is a key factor, but numerous studies lack detailed methods, allowing for integration into the estimation of development time when noting factors to consider for a posteriori temperature estimation [5]. The morphological and anatomical parameters observed with the naked eye rarely represent the actual age markers on statistical models of development to provide precise age calculation for PMI [6]. Therefore, more advanced methods are required to improve the accuracy of age estimation, not only for the post-feeding and pupal stages, but also for all developmental stages. Gene expression patterns potentially serve as a molecular tool for age estimation during postembryonic development of forensically important flies [7,8,9,10].

The family Sarcophagidae (known as flesh fly) comprises of approximately 3000 described species worldwide [11,12]. *Sarcophaga peregrina* (Robineau-Desvoidy, 1830) is one of the most common flesh flies, and it is widely spread from tropical to subtropical areas of Palaearctic, Oriental, and Oceanian regions [13]. The reproductive cycle of *S. peregrina* comprises three stages: larva, pupa, and adult. It is well-known for adopting the reproductive strategy of ovoviviparity (or ovolarviparity) [14]. The eggs are laid at an advanced stage of embryonic development and the larva emerges immediately after deposition. This type of reproduction reduces the stage of larval development (the time when eggs hatch into first instar larvae). *Sarcophaga peregrina* can be commonly found in insect succession patterns of human and animal corpses, and it is regarded as an important necrophagous flesh fly in forensic entomology [15,16,17]. *Sarcophaga peregrina* is also related to parasitic diseases and intestinal infectious diseases in humans and livestock, and it can cause myiasis [13,18,19]. Due to its medical and forensic significance, a comprehensive gene expression dataset was generated using RNA sequencing, and *de novo* assembly was performed to characterize the *S. peregrina* transcriptome [20]. In a previous study, we attempted to identify four genes (*Hsp90*, *A-alpha*, *AFP*, and *AFBP*) that exhibit developmental, stage-specific expression patterns during pupation [21]. The developmental patterns of this species were also investigated, which can be used as reliable indicators for PMI estimation [3]. In addition, pteridine fluorescence was applied to determine the age-dependent changes of adult *S. peregrina*, by exploiting differences in chemical components [22].

As a typical holometabolous fly, *S. peregrina* undergoes a complete metamorphosis [23], which is a complex process accompanied by drastic morphological and physiological changes [24]. It is the critical process during development in which the soft cuticle of a wandering larva is transformed into a hard puparium [25]. Specifically, the larval cuticle is degraded, and a new cuticle of the pupa is secreted by epidermal cells [26]. Juvenile hormone (JH) is an important hormone in holometabolous insects, which coordinates with 20-hydroxyecdysone (20E) to regulate growth and development [27,28]. In this study, in order to explore the molecular mechanism during metamorphosis and identify the specific genes that may serve as potential targets, transcriptome analysis was used to investigate gene expression dynamics from the larval to pupal stage of *S. peregrina*. In the previous study, we published the genome of *S. peregrina* for the first time and utilized the availability of annotated chromosome-level genome of *S. peregrina* (NCBI accession no. JABZEU000000000) as the reference genome for gene annotation [29]. The results obtained by the RNA-seq analysis were further confirmed using quantitative real-time PCR analysis (qRT-PCR), in order to discover the specific genes that may play a key role from the larval to pupal stages of *S. peregrina*.

## 2. Materials and Methods

### 2.1. Rearing and Sampling of S. peregrina

Adults of *S. peregrina* were trapped with pork liver bait in Changsha, Hunan Province, China, employing pork liver as a medium for larviposition and larval rearing. For this study, five lines were established and inbred for six generations to reduce genetic variability. In each generation, the mating pairs of adult *S. peregrina* from each line were kept at 25 ± 1 °C and 70 ± 5% relative humidity, with a photoperiod regime of 12:12 h light/darkness in an artificial climate chamber. Afterwards, the newly hatched larvae were continuously fed at 25 °C, until they emerged as adults. The sampled larvae were observed to determine the instar, based on the number of clefts in the posterior spiracle. The larval stage includes the first, second, and third instar. The third instar that jumped into sand were defined as being in the wandering (post-feeding) stage. The wandering larvae eventually reached metamorphosis, which was referred to as the pupal period (Figure S1). Larvae were collected every 4 h, until pupation. The pupal stage was observed every 8 h, until adult eclosion. The samples were frozen in liquid nitrogen and then stored at −80 °C. Three biological replicates were performed for all samples.

### 2.2. RNA-Seq and Data Analysis

A total of 150 specimens were collected, including hatched first instar larvae (*n* = 30, Sample O), as well as second instar larvae (*n* = 30, Sample B2), third instar larvae (*n* = 30, Sample B3), wandering larvae (*n* = 30, Sample B4), and pupae (*n* = 30, Sample B5). Total RNA was subsequently extracted using the mirVana miRNA Isolation kit (Ambion), following the manufacturer’s protocol. DNA contaminants were removed using DNase I (Promega, Madison, WI, USA). RNA integrity was evaluated using the Agilent 2100 Bioanalyzer (Agilent Technologies, Santa Clara, CA, USA). The libraries were constructed using TruSeq Stranded mRNA LTSample Prep kit (Illumina, San Diego, CA, USA), in accordance with the manufacturer’s instructions. Then, these libraries were sequenced on the Illumina sequencing platform (HiSeq 4000) (Illumina, Inc., San Diego, CA, USA), and 150 bp paired-end reads were generated in OE biotech Co., Ltd. (Shanghai, China). Raw reads were assessed for quality control using Trimmomatic [30] and then mapped to reference genome using HISAT 2.2 [31]. Only aligned reads were further analyzed, based on the reference genome [29]. The read counts of each gene were subsequently obtained by htseq-count [32], and the fragments per kb per million reads (FPKM) value of each gene was calculated using Cufflinks [33,34]. Furthermore, several databases were used to annotate gene functions: the Gene Ontology (GO, Kyoto Encyclopedia of Genes and Genomes (KEGG)) and Swiss-Prot/TrEMBL/InterPro databases.

Differentially expressed genes (DEGs) were identified using the estimateSizeFactors and nbinomTest functions, as implemented in the DESeq (2012) R package [35]. The *p* value of <0.05 and fold-change of >2 were set as the threshold to evaluate the significance of DEGs. A hierarchical cluster analysis of DEGs was performed to explore gene expression patterns. GO and KEGG enrichment analyses of DEGs were performed using the R package, based on hypergeometric distribution [36,37]. A gene set enrichment analysis (GSEA) was performed using a cluster profiler [38]. The clustering analysis of gene expression trends was performed using short time-series expression miner (STEM) v. 1.3.13 [39]. Additionally, in order to further cluster the genes with similar expression patterns across samples, a co-expression analysis was performed using a weighted co-expression network analysis (WGCNA) package [40]; then, the genes in the cluster were analyzed by KEGG and GO enrichment analysis.

### 2.3. qRT-PCR Analysis

Some of the most up- and down-regulated genes across the postembryonic development from the first larval instar to pupa were selected to validate the gene expression profiles by qRT-PCR analysis, including stable genes used as internal controls. In order to further predict the relationship between gene expression and developmental age in a blind study, larval samples were again collected using the above-described method. Total RNA was extracted and measured, and the methods were the same as those described above. RNA was reverse-transcribed using Goldenstara^TM^ RT6 Cdna Synthesis Mix (TSINGKE, Beijing, China), following the manufacturer’s protocol. Then, T5 Fast qPCR Mix (SYBR Green I) (TSINGKE, Beijing, China) was applied to qRT-PCR reactions with a 20 μL reaction volume, following the manufacturer’s protocol. The PCR reactions were performed with 1 min initial denaturation at 95 °C, followed by 40 cycles at 95 °C for 10 s and 60 °C for 15 s on an ABI7500 real-time PCR system (Applied Biosystems, Foster City, CA, USA). The specificity of the amplification products was evaluated using a 75 °C to 85 °C melting curve. Based on normalization with the reference genes, the 2^−ΔΔCT^ method was used to determine expression changes [41]. The relative mRNA levels of each gene were represented as folds over the expression levels of reference genes. All experiments were performed in three biological replicates.

### 2.4. Statistical Analysis

Based on the results of the qRT-PCR analysis mentioned above, five target genes were selected from the continuously up-regulated genes, as well as two stable reference genes. The samples were collected during the larval stage for further verification. One-way ANOVA was used to calculate the effect of different developmental times on the expression levels of target genes. Q–Q plots and Kolmogorov–Smirnov normality tests were used to determine the normal distribution of the log FC values. The relationship between the expression level and developmental times was analyzed to obtain equations of linear regression [21]. All statistical analyses were conducted with OriginPro v. 8.6, SPSS v. 22.0, and GraphPad Prism 6.

## 3. Results

### 3.1. Overview of the RNA-Seq Data

To investigate the gene expression profiles from the larval to pupal stage of *S. peregrina*, the purity and integrity of all RNA samples were assessed (Table S1), and then we performed RNA-seq analysis on five developmental stages. In total, 112.24 GB of raw data were obtained; 105.13 GB of clean data were retained after quality control, including an average of 48.91 MB clean reads from each sample. The data had an average GC content and Q30 of 41.84% and 93.75%, respectively (Table S2). Moreover, 90.44% to 96.48% of the clean reads could be mapped onto the assembled genome, and the percentage of unique mapping reads ranged from 81.60% to 90.38% (Table S3).

The FPKM was calculated and standardized for the analysis of gene expression, and similar patterns of FPKM density were obtained for each sample, suggesting the high reproducibility of transcriptomic analysis. In total, 12,169 genes were identified, and the gene expression profiles were shown (Table S4). Furthermore, in order to verify the reliability of the experimental design, we performed a correlation analysis of protein coding gene expression levels and principal component analysis (Figures S2 and S3), as well as the similarity analysis between samples (Figure S4).

### 3.2. Changes in Gene Expression Profiles during Developmental Stages

**Figure 1:**
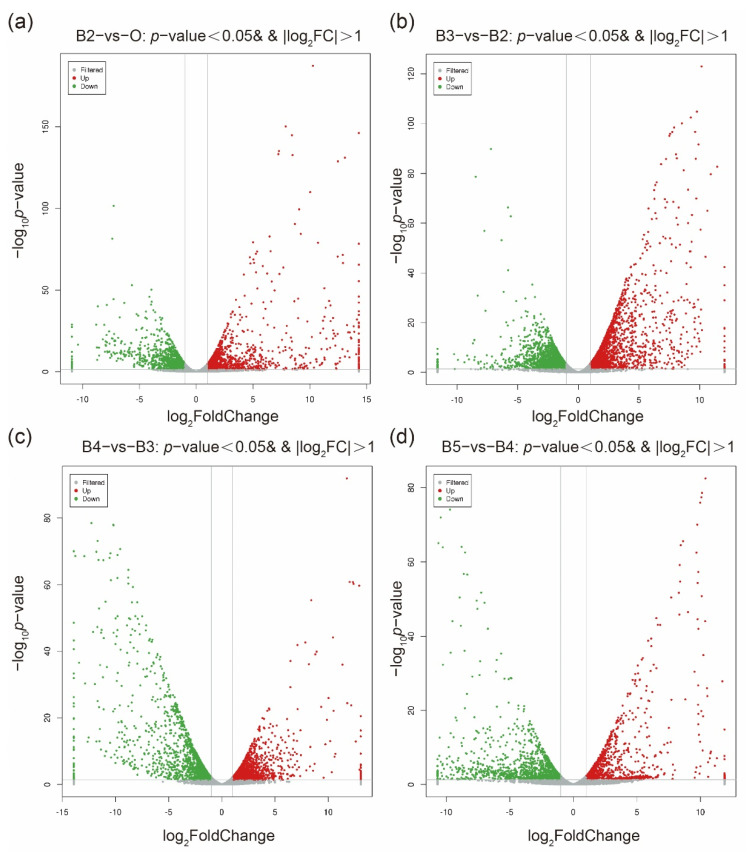
Development-associated transcriptomic profiles across the postembryonic development from the first larval instar to pupa of *S. peregrina*. In relation to the numbers of differentially expressed genes (DEGs), the up-regulated genes were represented by a red dot, and the down-regulated genes were represented by a blue dot. (**a**) B2 vs. O, (**b**) B3 vs. B2, (**c**) B4 vs. B3, (**d**) B5 vs. B4.

The count of genes was standardized in each sample using DESeq software. Significant DEGs were identified (*p* value < 0.05 and log_2_ FC > 1) during the developmental stages (Figure 1). A total of 2457 genes were identified, among which, 1329 were up-regulated and 1128 were down-regulated (B2 vs. 0). A total of 3656 genes were identified, of which, 1892 were up-regulated and 1764 were down-regulated (B3 vs. B2). A total of 3764 genes were identified as DEGs, with 1569 up-regulated and 2195 down-regulated (B4 vs. B3). A total of 2554 genes were identified as DEGs, with 1158 up-regulated and 1396 down-regulated (B5 vs. B4). To confirm whether genes clustering in the same branch have similar biological functions, a cluster analysis was performed on the selected DEGs to calculate the correlation between samples (Figure S5).

**Figure 2:**
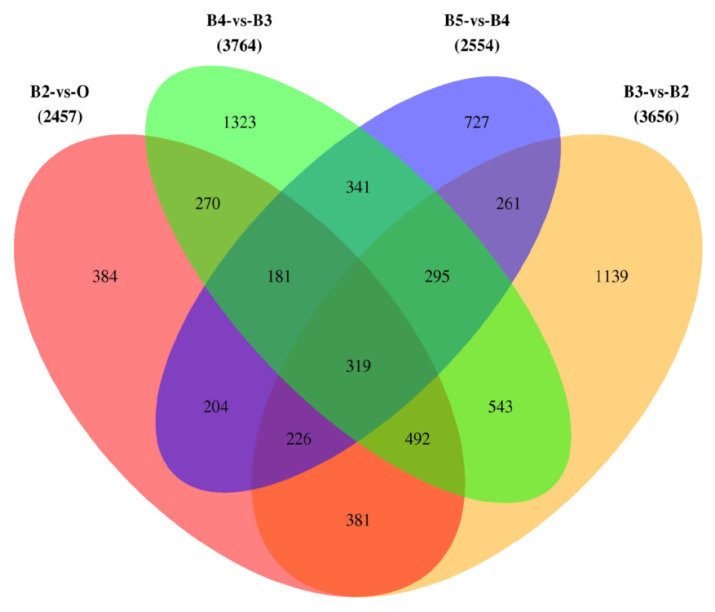
A Venn diagram showing the shared and unique genes between different groups.

**Figure 3:**
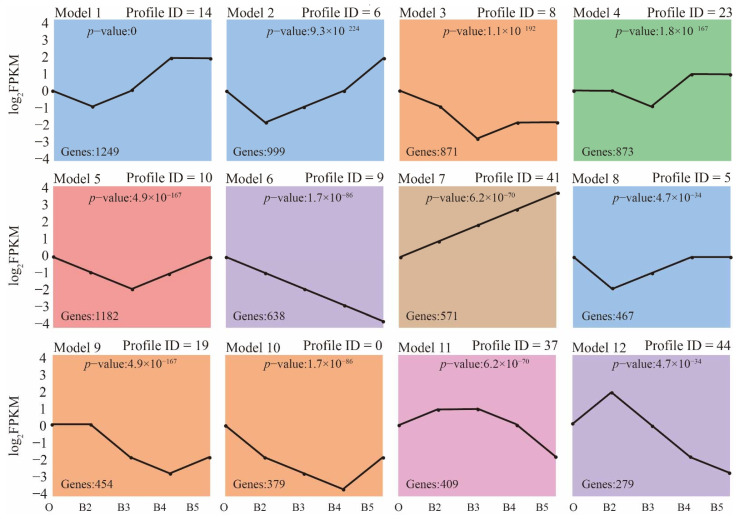
Series test of cluster (STC) analysis of DEGs during postembryonic development. The DEGs clustered into 12 major groups, based on patterns of gene expression during the five developmental stages using the STEM clustering method. The black line shows the average log_2_ FPKM to visualize the expression trend of each cluster. The same color indicates similar gene expression patterns. The *X* axis shows the five developmental stages. O: hatched first instar larvae, B2: second instar larvae, B3: third instar larvae, B4: wandering larvae and B5: pupae.

**Figure 4:**
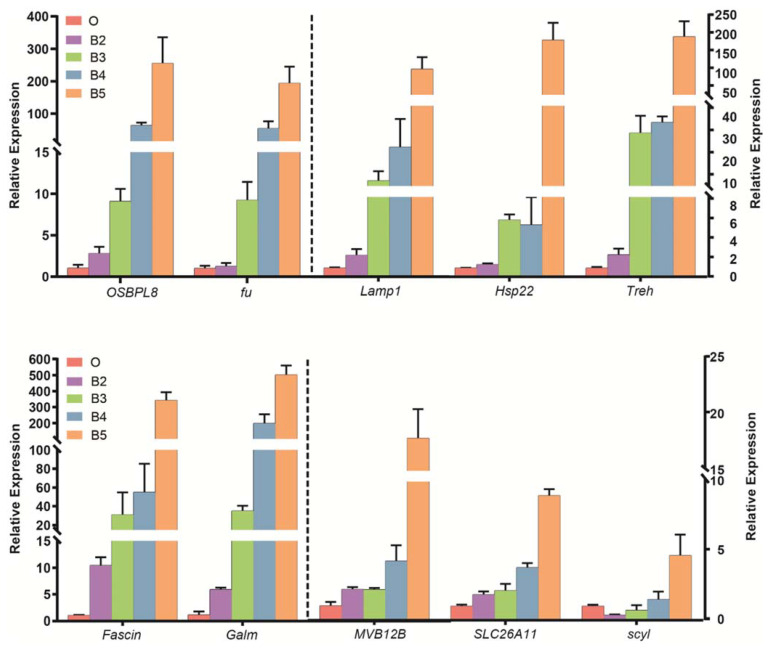
Expression of the selected 10 continually up-regulated DEGs by qRT-PCR. The data are shown as mean ± SE of three replications. O: the hatched first instar larvae, B2: the second instar larvae, B3: the third instar larvae, B4: the wandering larvae and B5: pupae. The left side of the dotted line shares the left *Y* axis, and the right side shares the right *Y* axis.

**Figure 5:**
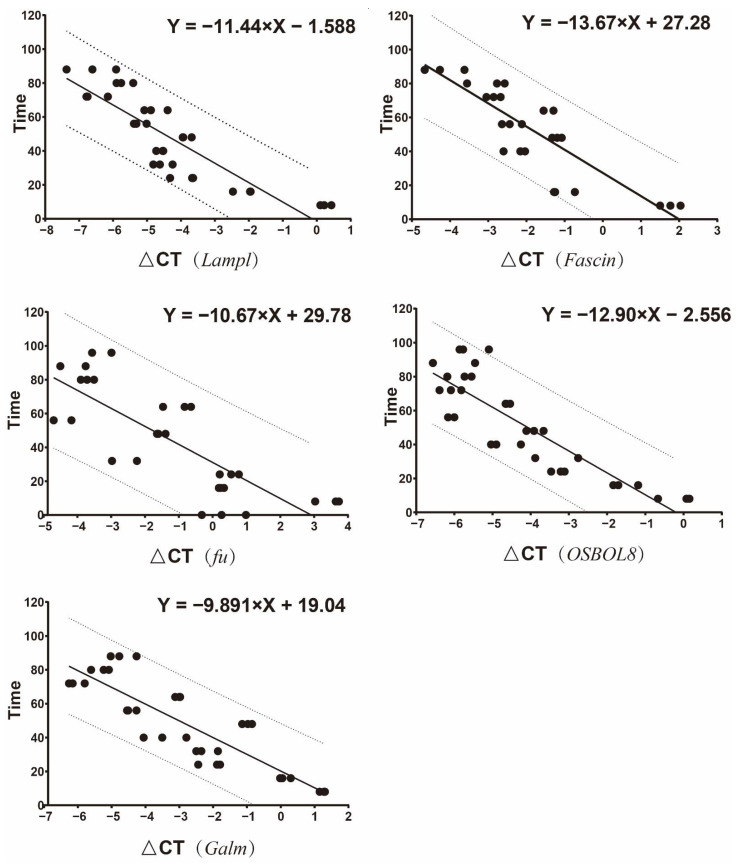
The linear regression equation between the relative expression levels of five target genes (*Fascin*, *Galm*, *Lamp1*, *OSBPL8*, and *fu*) and developmental time of larval stage. *Y* axis: sampling time (h); *X* axis: cycle threshold (CT) values of the target genes–the internal reference genes. The solid line is the linear regression equation, which denotes the predicted versus true development time, and the dotted lines denote predictions within 10% of true.

When the shared and unique genes of DEGs were statistically analyzed, there were 319 core DEGs. In the four comparisons, there were 384, 1139, 1323, and 727 unique DEGs (Figure 2). The expression patterns of all genes were then analyzed by performing the series test of cluster (STC) analysis. DEGs were significantly grouped into 12 clusters, based on expression profiles (Figure 3). Among the 12 clusters, 11 showed significant expression levels: second instar larvae (cluster 14/6/5/44), third instar larvae (cluster 8/10/23/37), and wandering larvae (cluster 18/0). In addition, two clusters (clusters 9 and 41) contained significantly expressed genes in the ‘↘’ type (638 genes) and ‘↗’ type (571 genes), indicating continuously down- and up-regulated expression patterns, respectively. Functional enrichment analysis of the genes within 12 clusters was further performed (Tables S5–S8). Moreover, these consistently up-regulated genes were further validated by qRT-PCR during postembryonic development. Finally, 10 DEGs were identified (*Fascin*, *Galm*, *Lamp1*, *Hsp22*, *Treh*, *OSBPL8*, *fu*, *MVB12B*, *SLC26A11*, and *scyl*), as well as two stably expressed genes (*PPP2R5E* and *ATP2B3*), selected as reference genes. The final primers used in this study are listed (Table S9). The qRT-PCR analysis was consistent with the results of comparative transcriptome analysis (Figure 4). In order to clarify the relationship between gene expression and age estimation, a total of 39 specimens during the larval stage of *S. peregrina* were collected in a blind study predicting developmental age (Table S10). The gene expression patterns of five target genes (*Fascin*, *Galm*, *Lamp1*, *OSBPL8*, and *fu*) were further verified using qRT-PCR. The relationship between gene expression and developmental times was established by the analysis of linear regression equation. The expression patterns were significantly upregulated with developmental times, indicating a significant correlation (Figure 5 and Table S11). On average, the estimated minimum age approximated the lower limit calculated by the regression equation, and the estimated maximum age approximated the calculated upper limit.

### 3.3. Gene Functional Annotation and Enrichment Analysis

Functional enrichment analyses were carried out in four comparisons. A total of 7409 and 3255 genes were successfully annotated in the GO and KEGG databases, respectively. The most enriched GO terms were ‘transaminase activity’ and ‘serine-type endopeptidase activity’, especially multiple DEGs encoding ‘the structural constituent of cuticle’ were significantly regulated from larval to pupal stage (Figure S6), suggesting degradation and synthesis of cuticle-related proteins actively occurring. After entering into the pupal stage, ‘serine-type endopeptidase inhibitor activity’ and ‘protein tyrosine kinase activity’ were significantly up-regulated, which may be related to melanin synthesis in the process of pupation. The KEGG enrichment analysis showed that 572, 938, 1098, and 575 DEGs were classified into nearly 45 pathways (Figure S7). The metabolism-related pathway was significantly enriched (Figure S8), such as some genes encoding the ‘protein processing in endoplasmic reticulum’ and ‘amino sugar and nucleotide sugar metabolism’ pathways, were significantly up-regulated; ‘thermogenesis’ and ‘fatty acid degradation’ were significantly down-regulated during the pupal stage, which is consistent with the previous study of decreased energy metabolism after pupation in *Drosophila melanogaster* (Meigen, 1830) [42].

**Figure 6:**
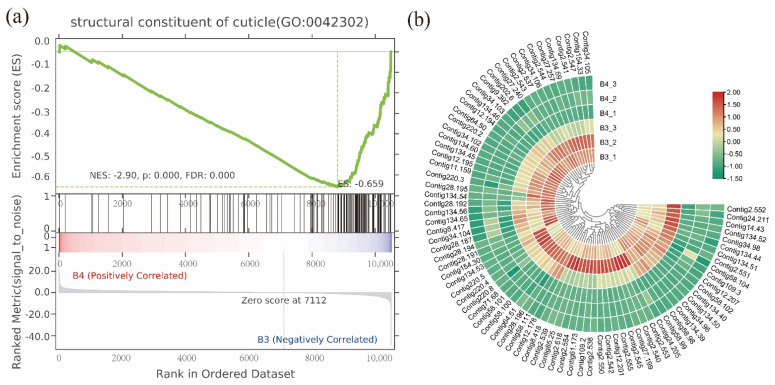
GSEA was performed to analyze the gene sets during postembryonic development. (**a**) GO terms showed that the structural constituent of cuticle was significantly up-regulated in the third instar (B4 vs. B3). (**b**) Expression profiles of genes associated with the structural constituent of cuticle. The color bar on the right shows the FPKM values from red (high) to green (low).

Furthermore, GSEA was performed to analyze the gene sets during postembryonic development. Data sets of the third instar, wandering, and pupal stage were selected, and then comparative analysis was performed. GO terms showed that ‘protein kinase activity’, ‘serine-type endopeptidase activity’, ‘oxidoreductase activity’, and ‘metabolic process’ were significantly enriched (B4 vs. B3). ‘Structural constituent of cuticle’ and ‘extracellular region’ were closely related to the synthesis and degradation of the cuticle (Figure 6 and Table S12). ‘Integral component of membrane’ was significantly up-regulated during pupation (B5 vs. B4).

**Figure 7:**
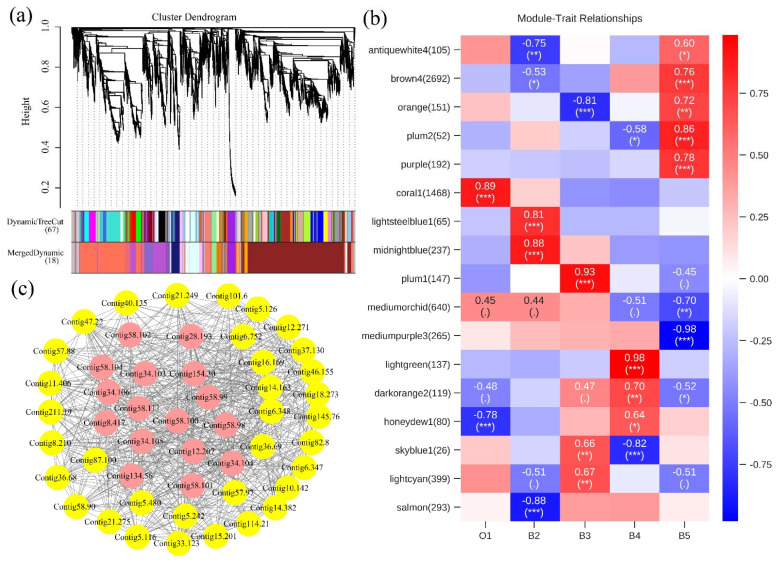
Hierarchical clustering dendrogram for modules identified by WGCNA. (**a**) Functional modules were shown with different colors. In total, 7206 genes were divided into 18 modules, which were assigned different colors. (**b**) Module-trait relationships. Each row corresponds to a module. Each column corresponds to a different developmental stage. The number of each cell at the row–column intersection indicates the correlation coefficient between the module and developmental stages; “.” indicates a *p* value greater than 0.1, “*” indicates a *p* value less than 0.1, “**” indicates a *p* value less than 0.01, “***” indicates a *p* value less than 0.001. The color bar on the right shows the correlation coefficient between traits and modular characteristic genes from red (high) to blue (low). (**c**) Weighted co-expression network for genes in the plum1 module. Yellow circles show top 50 hub genes in this module. Pink circles represent genes encoding cuticular proteins (*CPs*). Lines represent the weight of connectivity between genes.

To further identify the key genes during the five developmental stages, we analyzed the scale-free co-expression network and identified genetic modules that were associated with specific traits using WGCNA. Based on the correlation of the expression profiles among genes, we identified 18 modules, with the 18th module (grey) including genes that were not assigned to any module (Figure 7). Then, we characterized the gene expression pattern of each module based on the module eigengene. Each of 17 modules correlated with particular sample traits (Tables S13 and S14). The significantly correlated modules indicated a high expression pattern of specific traits. The functional enrichment analysis further revealed that the ‘structural constituent of cuticle’ (GO: 0042302) and ‘extracellular region’ (GO: 0005576) were significantly enriched in the plum1 module, which plays an important role in the synthesis and degradation of the cuticle [43]. The light green module was associated with ‘insect hormone biosynthesis’ (path: ko00981); it probably related to the 20E pathway during the wandering stage [44]. The plum2 module was associated with ‘tyrosine metabolism’ (path: ko00350), which might play a key role in the process of puparium tanning (sclerotization and pigmentation) [45].

### 3.4. Gene Expression Level Changes in 20-Hydroxyecdysone (20E) and Juvenile Hormone (JH) Pathways

**Figure 8:**
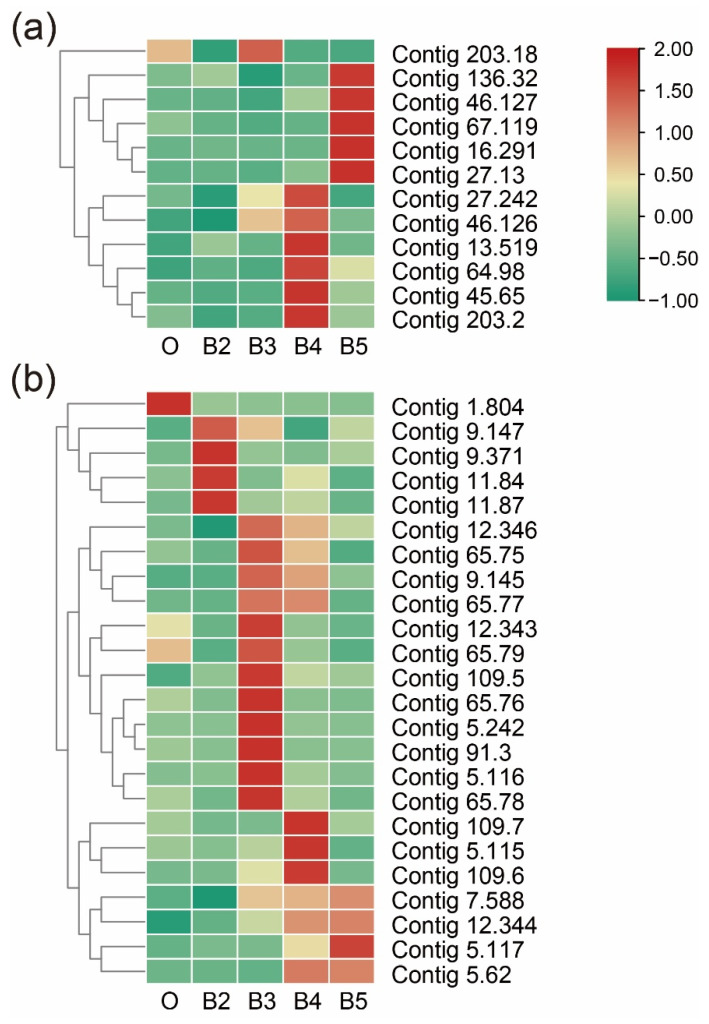
Gene expression profiles of the 20E and JH pathways. (**a**) Genes related to 20E synthesis. (**b**) Genes related to JH synthesis, degradation, and transport. The color scale on the right shows the FPKM values for each developmental stage from red (high) to green (low), standardized by min–max normalization.

From the larval to pupal stages, 12 genes showed different expression patterns in the 20E synthesis and signal pathways and were enriched in the “insect hormone biosynthesis” pathway for KEGG database, especially in “molting hormone (20E)”. Cholesterol 7-desaturase (*SPDAF36*) is a key enzyme in the initiation of the ecdysone synthesis, which was at a relatively high level in the first and third instars. Five genes encoding *SPImpE2*, *SPEip75B*, *SPEip78C*, and *SPecd* had high expression during pupation. The other five genes encoding *SPEcR*, *SPshd*, and *SPEip74EF* were highly expressed in the wandering stage. Additionally, four P450 genes encoding *SPCYP307a1/2*, *SPCYP302a1*, *SPCYP314a1*, and *SPCYP18a1* were significantly down-regulated during the pupal stage (Figure 8a).

The gene expression profiles of the JH pathway include JH synthesis, degradation, and transport (Figure 8b). For the JH synthesis pathway, the expression of the gene encoding juvenile hormone acid O-methyltransferase (*SPjhamt*) was significantly decreased during postembryonic development. The aldehyde dehydrogenase (*ALDH*) was significantly up-regulated from t he first to third instar stages, but significantly down-regulated from the wandering to pupal stages. For the JH degradation pathway, the gene expressions of JH epoxidation hydrolase 1 (*SPJHEH1b*) and *SPJHEH2* were significantly up-regulated at the larval stage and had relatively low levels during pupation. In addition, *SPJHEH1a* was continuously up-regulated during the developmental stages. The multiple (juvenile hormone binding protein) *SPJHBP* genes were identified in the JH transport pathway, and the expression levels of three genes were continuously up-regulated during postembryonic development, but the other genes were highly expressed during the larval stage, especially in the third instar.

### 3.5. Dynamic Expression Level Changes of Genes in Chitin-Related Pathway

Trehalase is a key enzyme in the initiation site of chitin synthesis pathway during postembryonic development. Genes encoding trehalase, trehalose-phosphate phosphatase, and facilitated trehalose transporter were identified in this study. The genes were significantly expressed at relatively high levels during the wandering stage, followed by the pupal stage. Genes encoding hexokinase, glucose-6-phosphate isomerase, glucosamine–fructose-6-phosphate, and glucosamine-6-phosphate N-acetyltransferase showed a dynamic expression pattern during developmental stages. The chitin synthetase was at a relatively high level from the larval-to-larval stages, and it was significantly down-regulated from the wandering to pupal stages. Furthermore, 42 differentially expressed genes were identified in the chitin-binding domain superfamily. Most genes were continuously expressed at high levels in the third instar and significantly lower in the pupal stage. However, the expression of five genes (id: Contig37.24, Contig2.438, Contig105.36, Contig72.89, and Contig67.103) were significantly up-regulated during postembryonic development.

**Figure 9:**
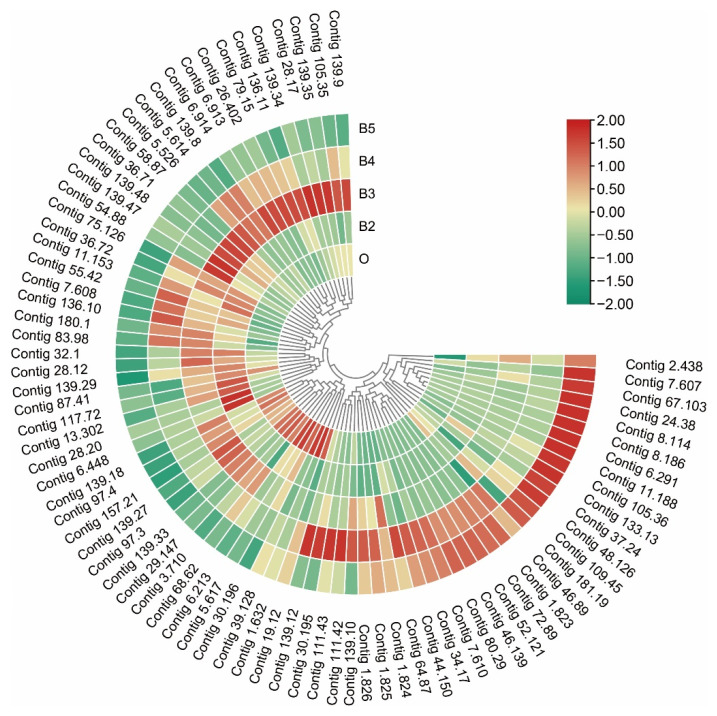
Expression profiles of genes in chitin-related pathway. The color scale on the right shows the FPKM values for each developmental stage from red (high) to green (low), standardized by min–max normalization.

For the chitin degradation pathway, the mRNA level for encoding β-N-acetylglucosaminidase (id: Contig46.139, Contig24.38, Contig52.121, and Contig48.126) was significantly up-regulated during postembryonic development. The chitin deacetylase (Contig139.47 and Contig139.48) was continuously up-regulated from the first instar to the third instar and down-regulated during pupation. Genes encoding the *SPIdgf5* and *SPIdgf1* were significantly up-regulated from the first to third instars and continuously down-regulated during pupation. The expression levels of *SPIdgf1* and *SPChia* were continuously up-regulated during the developmental processes, and *SPthbs3b* and *SPCht10b* were highest during the pupal stage (Figure 9).

### 3.6. Dynamic Expression in Cuticle-Related Genes

**Figure 10:**
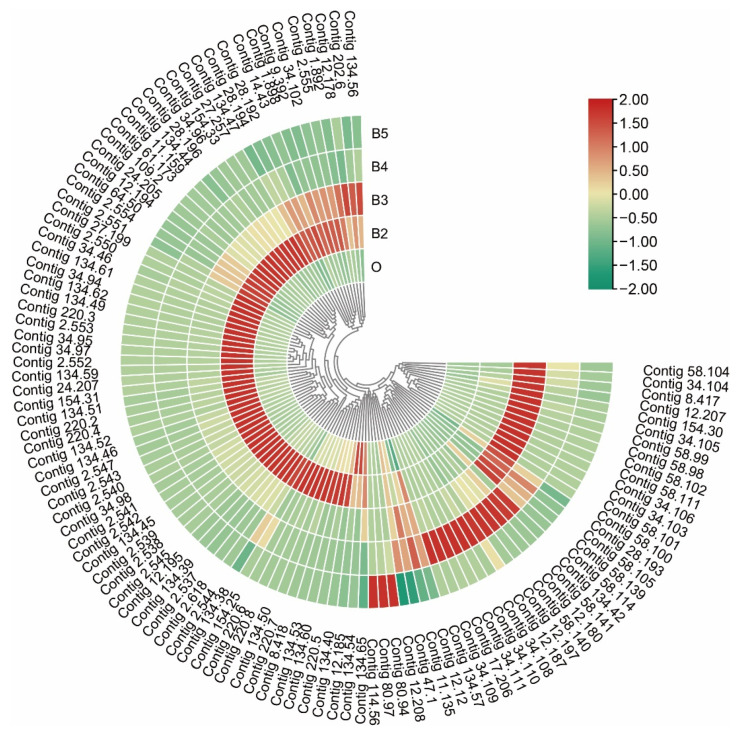
Expression profiles of cuticular protein genes, including the families encoding cuticle protein, larval cuticle protein, pupal cuticle protein, adult-specific cuticular protein, and endocuticle structural glycoprotein. The color scale on the right shows the FPKM values for each developmental stage from red (high) to green (low), standardized by min–max normalization.

The genes encoding cuticular protein were identified during developmental stages, including cuticle proteins (*CPs*), larval cuticle proteins (*LCPs*), pupal cuticle proteins (*PCPs*), adult-specific cuticular proteins (*ACPs*), and endocuticle structural glycoprotein (*SgAbd*). Five genes encoding endocuticle structural glycoprotein (*SgAbd-9*) had obviously different expression patterns. Two genes (id: Contig114.56 and Contig80.94) were continuously up-regulated during postembryonic development. Two genes encoding *SgAbd-2/3* were significantly up-regulated from the first to third instars and continuously down-regulated during pupation. The gene families (*SPCP*, *SPLCP2*, *SPLCPA2B*, *SPLCPA3A*, *SPLCP8*, *SPACP1*, *SPACP20*, and *SPPCPEdg-84A*) were highly expressed in the second instar and significantly down-regulated during pupation. Four genes encoding *SPLCP4* (id: Contig34.111 and Contig34.110) and *SPLCP2* (id: Contig34.108 and Contig34.109) were expressed at a very high level in the wandering stage (Figure 10).

## 4. Discussion

A precise age calculation of the immature insects commonly colonized on decomposed corpses has great potential to improve PMI estimation, based on entomological evidence [9]. To date, morphological and genetic markers have been identified as effective methods to divide the metamorphosis process of insects into different developmental periods [3,21]. Due to some limitations and difficulties of morphological identification in the age estimation of immature flies, especially in the post-feeding larvae and pupae [3,4], the temporal gene expression patterns indicated that they can be utilized for age estimation during the life cycle of *D. melanogaster* [46]. In recent years, the analysis of differentially expressed genes has been reported as a useful diagnostic tool for forensic entomology; seven genes (*chitin synthase*, *ecdysone receptor*, *hsp60*, *hsp90*, *rop-1*, *ultraspiracle*, and *white*) were differentially regulated during all developmental stages of *Lucilia sericata* (Meigen, 1826), which can be more accurately used for age prediction, i.e., for the wandering larval and pupal stages, in particular [10]. Additionally, gene expression patterns (*EcdR*, *AR*, and *15_2*) during the larval development of *Calliphora vicina* (Robineau-Desvoidy, 1830) showed reliable tendencies to identify the post-feeding stage more precisely [7]. The analysis of temporally regulated genes also proved suitable for age prediction in *C. vicina* pupae and as a diagnosis tool to recognize diapause in post-feeding larvae [8,47,48]. Afterwards, a *de novo* transcriptome analysis was performed at 15 different pupal stages of *C. vicina*, which were used to identify sensitive gene expression profiles as candidates for the molecular age estimation of pupae [9]. However, identification of appropriate genetic markers, and the application of this methodology to the age determination of necrophagous insects is still in infancy [8].

Although previous studies have verified that the analysis of gene expression patterns can assist in age determination in *S. peregrina* [20,21], both target and reference genes were selected from other species and then repeatedly validated, with some limitations. Due to a lack of complete reference genome and low homology between this species and the *D. melanogaster*, the potential function of these genes remains unclear in *S. peregrina*. The aim of this study was to identify highly specific genetic markers, whose expression profiles would predict age estimation from the larval to pupal stages of *S. peregrina*. On the basis of previous studies [20,21,29], we analyzed the transcriptome of different developmental stages; a total of 12,169 genes were identified, based on the published genome, as a reference in this study. Based on normalization with two reference genes, the gene expression patterns of 10 target genes were further verified and significantly upregulated with developmental time. The differentially expressed genes were then examined in a blind study, indicating the ability to predict the larval development. The developmental, stage-specific gene profiles could suggest the novel molecular biomarkers for different developmental stages of forensically important flies. Nonetheless, aging (i.e., the processing of all developmental stages) relies on temporal gene expression patterns during all developmental stages and is mainly regulated by hormones [23]. Therefore, studies on the molecular mechanism during metamorphosis can provide powerful evidence for identifying the specific genes that may serve as potential targets.

Generally, the cuticle of an insect provides mechanical support and acts as an effective barrier to protect against physical and chemical damage, pathogen invasion, and water loss [49]. Degradation of the larval cuticle and regeneration of the pupal cuticle are two successive processes during postembryonic development [23,24]. Chitin is one of the major components of cuticle. The chitin biosynthetic pathway consists of eight enzyme-catalyzed reactions, starting with trehalase and ending with the chitin synthase [43]. As expected, the present study demonstrated that the genes involving chitin biosynthesis pathway, hexokinase, glucose-6-phosphate isomerase, glucosamine–fructose-6-phosphate, glucosamine-6-phosphate N-acetyltransferase, chitin synthetase, and the chitin-binding domain superfamily were significantly up-regulated during larval molting, suggesting their role in the chitin synthesis. Trehalase is associated with insect physiology as it regulates growth and energy metabolism [50]. Trehalase and a facilitated trehalose transporter were differentially expressed at relatively high levels from the larval to wandering stages of *S. peregrina*.

For the chitin degradation pathway, the expression levels of three main degrading enzymes (chitinase, β-N-acetylglucosaminidase and chitin deacetylase) were significantly up-regulated and at a relatively high level during pupation. The gene expression of chitin deacetylase and chitinase-like protein *Idgf1b/5* were continuously up-regulated from the first to third instar stages and down-regulated during pupation, supporting a pivotal role in the degradation of larval cuticle. A previous study showed that injection of dsRNAs for chitin deacetylase affected larval adult moltings in the red flour beetle (*Tribolium castaneum*) [51]. However, β-N-acetylglucosaminidase, endochitinase, and chitinase-like protein *Idgf1a* were significantly up-regulated during the developmental stages, suggesting that they play an important role during metamorphosis [52,53]. When larvae enter pupation, the cuticle proteins are broken down, and the chitin is released [43]. In this study, five to eight cuticle-related (including chitin-related and *CPs*) GO terms (0042302: structural constituent of cuticle) were significantly enriched during metamorphosis. The expression levels of encoding *SPCP*, *SPLCP2*, and *SPLCP8* family were at a relatively higher level in the second instar, and they were down-regulated during pupation. The genes of *SPLCP5* were significantly up-regulated from the first to third instar stages. In brief, the expression profiles of genes encoding enzymes involved in chitin metabolism showed dynamic changes associated with the processes of larval molting [43,54].

The hormones JH and 20E regulate the biological processes of growth, molting, metamorphosis, and reproduction of insects [55,56]. The 20E is the ecdysis hormone, which can trigger the ecdysis process and mediate morphogenetic processes during metamorphosis [57]. The 20E signal pathway is composed of ecdysone-induced proteins (*E74*, *E75*, and *E78*) that can strictly control the metamorphosis of insects [52]. The specific *P450* genes (*CYP302a1*, *CYP307a1*, and *CYP314a1*) are also involved in the 20E biosynthetic pathway. It was found that the RNAi-mediated knockdown of *CYP307a1* (*SPO*) reduced the level of 20E, caused precocious metamorphosis, and inhibited the emergence of adultoids in *Schistocerca gregaria* (Forskål, 1775) [58,59]. In the present study, the genes encoding 20E biosynthesis and the signaling pathway were significantly expressed at high levels in the wandering and pupal stages. This study confirmed that the high expression of these genes is essential for the initiation of the pupal process.

The JH is involved in the regulation of insect molting and metamorphosis by modulating ecdysone action [60,61]. Previous studies have revealed that changes of JH titers are primarily controlled through the JH synthesis and degradation pathways by the action of specific enzymes [62]. Numerous proteins involved in regulating JH biosynthesis have been identified [55]. However, only three kinds of enzymes, i.e., JH esterase, JHEH, and JH diol kinase, have been identified as participating in JH metabolism [63]. In the present study, the genes encoding *JHEH* were significantly up-regulated during the larval stage, but had relatively low levels during pupation. Furthermore, *JHBP* could serve as a carrier to bind the JH in the hemolymph and release the hormone to target tissues [60]. As expected, the JH transport pathway was significantly expressed at a high level during the larval stage, with relatively low expression level during pupation. The results suggest that 20E and JH regulate metamorphosis via the regulation of specific genes involved in the biosynthesis and signaling pathways. Besides, the larval stage is the critical period to obtain energy to metamorphosis into pupa [42,64]. Previous studies have reported that energy metabolic rates declined sharply after pupation and are maintained at a low level until eclosion [64,65]. As expected, the KEGG analysis in this study showed that the oxidative phosphorylation pathway was significantly down-regulated after molting into the wandering stage and less energy was required during the transition from third larval instar to the pupa than in the larval stage [42].

## 5. Conclusions

In this study, comprehensive transcriptomic analysis explored the importance of genetic markers that can serve as potential targets for age estimation from the larval to pupal stages of *S. peregrina*. These results provide a basis for establishing molecular age determination of *S. peregrina*, as well as other forensically important flies. Moreover, the study provides insights into molecular developmental biology during postembryonic development. Many genes involved in the structural constituent of cuticle, chitin synthesis, degradation pathways, and cuticular proteins were identified. The specific genes in the 20E and JH pathways were dynamically regulated, suggesting the important role in metamorphosis-related processes. WGCNA demonstrated the potential regulation mechanism of tyrosine metabolism in the process of puparium tanning. As a next step, the validation of these developmental, stage-specific genetic markers is to be performed. In order to refine the age estimation of the species, the gene expression profiles of each developmental stage require further analysis.

## Data Availability

Raw sequencing data of transcriptome have been deposited in the SRA (Sequence Read Archive) database of NCBI (National Center for Biotechnology Information) via accession numbers SRR9722714, SRR9722715, and SRR9722721, with the Bioproject ID PRJNA509973, as well as (SRR14699492–SRR14699494 and SRR14710521–SRR14710529), with the Bioproject ID PRJNA733571.

## Supplementary Materials

The following supporting information can be downloaded at: https://www.mdpi.com/article/10.3390/insects13050453/s1, Figure S1: The life cycle of *S. peregrina*; Figure S2: Clustering analysis between samples; Figure S3: PCA based on the expression level of genes; Figure S4: Similarity analysis between samples (Heat map); Figure S5: Heat map of differentially expressed genes (DEGs); Figure S6: GO significant enrichment analysis for differentially expressed genes (DEGs) during postembryonic development, (a) B2 vs. O, (b) B3 vs. B2, (c) B4 vs. B3, (d) B5 vs. B4; Figure S7: KEGG enrichment analysis of DEGs during postembryonic development. The numbers above bars represent the number of enriched genes, (a) B2 vs. O, (b) B3 vs. B2, (c) B4 vs. B3, (d) B5 vs. B4; Figure S8: KEGG significant enrichment analysis for differentially expressed genes (DEGs) during postembryonic development, (a) B2 vs. O, (b) B3 vs. B2, (c) B4 vs. B3, (d) B5 vs. B4; Table S1: The purity and integrity of all RNA samples were assessed; Table S2: Summary statistics of raw transcriptome data during developmental stages; Table S3: Data statistic of original transcripts aligned with genome assembly; Table S4: Statistic and annotation of gene expression profiles (FPKM); Table S5: GO significant enrichment analysis of continually down-regulated genes; Table S6: KEGG significant enrichment analysis of continually down-regulated genes; Table S7: GO significant enrichment analysis of continually up-regulated genes; Table S8: KEGG significant enrichment analysis of continually up-regulated genes; Table S9: Primers used in this study for qRT-PCR analysis; Table S10: Samples were collected for use in a blind study predicting developmental age; Table S11: The linear regression equation between the expression level of differential genes and development time; Table S12: GO significant enrichment analysis of gene set (B4 vs. B3); Table S13: The correlation coefficient between the module and developmental stage; Table S14: *p*-Value of The correlation coefficient between the module and traits.

## References

[1] Tomberlin J.K., Mohr R., Benbow M.E., Tarone A.M., VanLaerhoven S. (2011). A roadmap for bridging basic and applied research in forensic entomology. Annu. Rev. Entomol..

[2] Grassberger M., Reiter C. (2001). Effect of temperature on *Lucilia sericata* (Diptera: Calliphoridae) development with special reference to the isomegalen- and isomorphen-diagram. Forensic Sci. Int..

[3] Wang Y., Wang J.F., Zhang Y.N., Tao L.Y., Wang M. (2017). Forensically important *Sarcophaga*
*peregrina* (Diptera: Sarcophagidae) in China: Development pattern and significance for estimating postmortem interval. J. Med. Entomol..

[4] Baqué M., Filmann N., Verhoff M.A., Amendt J. (2015). Establishment of developmental charts for the larvae of the blow fly *Calliphora vicina* using quantile regression. Forensic Sci. Int..

[5] Charabidze D., Hedouin V. (2019). Temperature: The weak point of forensic entomology. Int J. Leg. Med..

[6] Matuszewski S. (2021). Post-mortem interval estimation based on insect evidence: Current challenges. Insects.

[7] Baqué M., Amendt J., Verhoff M.A., Zehner R. (2015). Descriptive analyses of differentially expressed genes during larval development of *Calliphora vicina* (Diptera: Calliphoridae). Int. J. Leg. Med..

[8] Boehme P., Spahn P., Amendt J., Zehner R. (2014). The analysis of temporal gene expression to estimate the age of forensically important blow fly pupae: Results from three blind studies. Int. J. Leg. Med..

[9] Zajac B.K., Amendt J., Horres R., Verhoff M.A., Zehner R. (2015). De novo transcriptome analysis and highly sensitive digital gene expression profiling of *Calliphora vicina* (Diptera: Calliphoridae) pupae using MACE (Massive Analysis of cDNA Ends). Forensic Sci. Int. Genet..

[10] Tarone A.M., Foran D.R. (2011). Gene expression during blow fly development: Improving the precision of age estimates in forensic entomology. J. Forensic Sci..

[11] Pape T. (1996). Catalogue of the Sarcophagidae of the world (Insecta: Diptera). Mem. Entomol. Int..

[12] Buenaventura E., Szpila K., Cassel B.K., Wiegmann B.M., Pape T. (2020). Anchored hybrid enrichment challenges the traditional classification of flesh flies (Diptera: Sarcophagidae). Syst. Entomol..

[13] Xue W.Q., Verves Y.G., Du J. (2011). A review of subtribe *Boettcheriscina* Verves 1990 (Diptera: Sarcophagidae), with descriptions of a new species and genus from China. Ann. Soc. Ent. Fr..

[14] Majumder M.Z.R., Dash M.K., Khan H.R., Khan R.A. (2014). The reproductive biology of flesh fly, *Sarcophaga peregrina* (Robineau-Desvoidy, 1830) (Diptera: Sarcophagidae). Dhaka Univ. J. Biol. Sci..

[15] Wang Y., Ma M.Y., Jiang X.Y., Wang J.F., Li L.L., Yin X.J., Wang M., Lai Y., Tao L.Y. (2017). Insect succession on remains of human and animals in Shenzhen, China. Forensic Sci. Int..

[16] Sukontason K., Bunchu N., Chaiwong T., Moophayak K., Sukontason K.L. (2010). Forensically important flesh fly species in Thailand: Morphology and developmental rate. Parasitol. Res..

[17] Toukairin Y., Arai T., Hoshi T., Oliva Trejo J.A., Nogami M. (2017). The geographical distribution of fly larvae on corpses in Saitama Prefecture in Japan during the summer season. Leg. Med..

[18] Lee Y.T., Chen T.L., Lin Y.C., Fung C.P., Cho W.L. (2011). Nosocomial nasal myiasis in an intubated patient. J. Chin. Med. Assoc..

[19] Miura M., Hayasaka S., Yamada T., Hayasaka Y., Kamimura K. (2005). Ophthalmomyiasis caused by larvae of *Sarcophaga peregrina*. Jpn. J. Ophthalmol..

[20] Kim J.Y., Lim H.Y., Shin S.E., Cha H.K., Seo J.H., Kim S.K., Park S.H., Son G.H. (2018). Comprehensive transcriptome analysis of *Sarcophaga peregrina*, a forensically important fly species. Sci. Data.

[21] Shang Y., Ren L., Yang L., Wang S., Chen W., Dong J., Ma H., Qi X., Guo Y. (2020). Differential gene expression for age estimation of forensically important *Sarcophaga peregrina* (Diptera: Sarcophagidae) intrapuparial. J. Med. Entomol..

[22] Zhu G.H., Ye G.Y., Li K., Hu C., Xu X.H. (2013). Determining the age of adult flesh flies, *Sarcophaga peregrina*, using pteridine fluorescence. Med. Vet. Entomol..

[23] Rolff J., Johnston P.R., Reynolds S. (2019). Complete metamorphosis of insects. Philos. Trans. R. Soc. Lond. B Biol. Sci..

[24] Truman J.W., Riddiford L.M. (1999). The origins of insect metamorphosis. Nature.

[25] Nachman R.J., Strey A., Zubrzak P., Zdarek J. (2006). A comparison of the pupariation acceleration activity of pyrokinin-like peptides native to the flesh fly: Which peptide represents the primary pupariation factor?. Peptides.

[26] Zhu K.Y., Merzendorfer H., Zhang W., Zhang J., Muthukrishnan S. (2016). Biosynthesis, turnover, and functions of chitin in insects. Annu. Rev. Entomol..

[27] Santos C.G., Humann F.C., Hartfelder K. (2019). Juvenile hormone signaling in insect oogenesis. Curr. Opin. Insect Sci..

[28] Luo W., Veeran S., Wang J., Li S., Li K., Liu S.N. (2020). Dual roles of juvenile hormone signaling during early oogenesis in *Drosophila*. Insect Sci..

[29] Ren L., Shang Y., Yang L., Wang S., Wang X., Chen S., Bao Z., An D., Meng F., Cai J. (2021). Chromosome-level *de novo* genome assembly of *Sarcophaga peregrina* provides insights into the evolutionary adaptation of flesh flies. Mol. Ecol. Resour..

[30] Bolger A.M., Lohse M., Usadel B. (2014). Trimmomatic: A flexible trimmer for Illumina sequence data. Bioinformatics.

[31] Kim D., Langmead B., Salzberg S.L. (2015). HISAT: A fast spliced aligner with low memory requirements. Nat. Methods.

[32] Anders S., Pyl P.T., Huber W. (2015). HTSeq—A Python framework to work with high-throughput sequencing data. Bioinformatics.

[33] Roberts A., Trapnell C., Donaghey J., Rinn J.L., Pachter L. (2011). Improving RNA-Seq expression estimates by correcting for fragment bias. Genome Biol..

[34] Trapnell C., Williams B.A., Pertea G., Mortazavi A., Kwan G., van Baren M.J., Salzberg S.L., Wold B.J., Pachter L. (2010). Transcript assembly and quantification by RNA-Seq reveals unannotated transcripts and isoform switching during cell differentiation. Nat. Biotechnol..

[35] Anders S., Huber W. (2010). Differential expression analysis for sequence count data. Genome Biol..

[36] Young A., Whitehouse N., Cho J., Shaw C. (2005). OntologyTraverser: An R package for GO analysis. Bioinformatics.

[37] Kanehisa M., Araki M., Goto S., Hattori M., Hirakawa M., Itoh M., Katayama T., Kawashima S., Okuda S., Tokimatsu T. (2008). KEGG for linking genomes to life and the environment. Nucleic Acids Res..

[38] Subramanian A., Tamayo P., Mootha V.K., Mukherjee S., Ebert B.L., Gillette M.A., Paulovich A., Pomeroy S.L., Golub T.R., Lander E.S. (2005). Gene set enrichment analysis: A knowledge-based approach for interpreting genome-wide expression profiles. Proc. Natl. Acad. Sci. USA.

[39] Ernst J., Bar-Joseph Z. (2006). STEM: A tool for the analysis of short time series gene expression data. BMC Bioinform..

[40] Langfelder P., Horvath S. (2008). WGCNA: An R package for weighted correlation network analysis. BMC Bioinform..

[41] Livak K.J., Schmittgen T.D. (2001). Analysis of relative gene expression data using real-time quantitative PCR and the 2(-Delta Delta C(T)) Method. Methods.

[42] Merkey A.B., Wong C.K., Hoshizaki D.K., Gibbs A.G. (2011). Energetics of metamorphosis in *Drosophila melanogaster*. J. Insect Physiol..

[43] Merzendorfer H., Zimoch L. (2003). Chitin metabolism in insects: Structure, function and regulation of chitin synthases and chitinases. J. Exp. Biol..

[44] Grau V., Lafont R. (1994). Metabolism of ecdysone and 20-hydroxyecdysone in adult *Drosophila melanogaster*. Insect Biochem. Mol. Biol..

[45] Chen E.H., Hou Q.L., Wei D.D., Dou W., Liu Z., Yang P.J., Smagghe G., Wang J.J. (2018). Tyrosine hydroxylase coordinates larval-pupal tanning and immunity in oriental fruit fly (*Bactrocera dorsalis*). Pest Manag. Sci..

[46] Arbeitman M.N., Furlong E.E., Imam F., Johnson E., Null B.H., Baker B.S., Krasnow M.A., Scott M.P., Davis R.W., White K.P. (2002). Gene expression during the life cycle of *Drosophila melanogaster*. Science.

[47] Fremdt H., Amendt J., Zehner R. (2014). Diapause-specific gene expression in *Calliphora vicina* (Diptera: Calliphoridae)—A useful diagnostic tool for forensic entomology. Int. J. Leg. Med..

[48] Boehme P., Spahn P., Amendt J., Zehner R. (2013). Differential gene expression during metamorphosis: A promising approach for age estimation of forensically important *Calliphora vicina* pupae (Diptera: Calliphoridae). Int. J. Leg. Med..

[49] Vincent J.F., Wegst U.G. (2004). Design and mechanical properties of insect cuticle. Arthropod. Struct. Dev..

[50] Shukla E., Thorat L.J., Nath B.B., Gaikwad S.M. (2015). Insect trehalase: Physiological significance and potential applications. Glycobiology.

[51] Arakane Y., Dixit R., Begum K., Park Y., Specht C.A., Merzendorfer H., Kramer K.J., Muthukrishnan S., Beeman R.W. (2009). Analysis of functions of the chitin deacetylase gene family in *Tribolium castaneum*. Insect Biochem. Mol. Biol..

[52] Gu J., Huang L.X., Gong Y.J., Zheng S.C., Liu L., Huang L.H., Feng Q.L. (2013). *De novo* characterization of transcriptome and gene expression dynamics in epidermis during the larval-pupal metamorphosis of common cutworm. Insect Biochem. Mol. Biol..

[53] Chen E.H., Hou Q.L., Dou W., Wei D.D., Yue Y., Yang R.L., Yu S.F., De Schutter K., Smagghe G., Wang J.J. (2018). RNA-seq analysis of gene expression changes during pupariation in *Bactrocera dorsalis* (Hendel) (Diptera: Tephritidae). BMC Genom..

[54] Liu S.H., Xia Y.D., Zhang Q., Li W., Li R.Y., Liu Y., Chen E.H., Dou W., Stelinski L.L., Wang J.J. (2020). Potential targets for controlling *Bactrocera dorsalis* using cuticle- and hormone-related genes revealed by a developmental transcriptome analysis. Pest Manag. Sci..

[55] Bellés X., Martín D., Piulachs M.D. (2005). The mevalonate pathway and the synthesis of juvenile hormone in insects. Annu. Rev. Entomol..

[56] Riddiford L.M., Truman J.W., Mirth C.K., Shen Y.C. (2010). A role for juvenile hormone in the prepupal development of *Drosophila melanogaster*. Development.

[57] Petryk A., Warren J.T., Marqués G., Jarcho M.P., Gilbert L.I., Kahler J., Parvy J.P., Li Y., Dauphin-Villemant C., O’Connor M.B. (2003). Shade is the *Drosophila* P450 enzyme that mediates the hydroxylation of ecdysone to the steroid insect molting hormone 20-hydroxyecdysone. Proc. Natl. Acad. Sci. USA.

[58] Cabrera A.R., Shirk P.D., Evans J.D., Hung K., Sims J., Alborn H., Teal P.E. (2015). Three Halloween genes from the Varroa mite, Varroa destructor (Anderson & Trueman) and their expression during reproduction. Insect Mol. Biol..

[59] Sugahara R., Tanaka S., Shiotsuki T. (2017). RNAi-mediated knockdown of SPOOK reduces ecdysteroid titers and causes precocious metamorphosis in the desert locust *Schistocerca gregaria*. Dev. Biol..

[60] Jindra M., Bittova L. (2020). The juvenile hormone receptor as a target of juvenoid “insect growth regulators”. Arch. Insect Biochem. Physiol..

[61] Jindra M., Palli S.R., Riddiford L.M. (2013). The juvenile hormone signaling pathway in insect development. Annu. Rev. Entomol..

[62] Li K., Jia Q.Q., Li S. (2019). Juvenile hormone signaling—A mini review. Insect Sci..

[63] Yin Y., Qiu Y.W., Huang J., Tobe S.S., Chen S.S., Kai Z.P. (2020). Enzymes in the juvenile hormone biosynthetic pathway can be potential targets for pest control. Pest Manag. Sci..

[64] Chamberlin M.E. (2004). Control of oxidative phosphorylation during insect metamorphosis. Am. J. Physiol. Regul. Integr. Comp. Physiol..

[65] Kaczmarek A., Wrońska A.K., Kazek M., Boguś M.I. (2020). Metamorphosis-related changes in the free fatty acid profiles of *Sarcophaga* (*Liopygia*) *argyrostoma* (Robineau-Desvoidy, 1830). Sci. Rep..

