# Supplementary material for: Temporal Expression Profiles Reveal Potential Targets during Postembryonic Development of Forensically Important Sarcophaga peregrina (Diptera: Sarcophagidae)
Source: Insects. 2022 May 12;13(5):453. doi: 10.3390/insects13050453 (PMC9143129; doi:10.3390/insects13050453)
Supplement: Supplementary file 1 [file insects-13-00453-s001.zip › Supplementary Figures.pdf]

Temporal expression profiles reveal potential targets during postembryonic development of forensically important *Sarcophaga peregrina* (Diptera: Sarcophagidae)

Figure S1. The life cycle of *S. peregrina*.

Figure S2. Clustering analysis between samples.

Figure S3. PCA based on the expression level of genes.

Figure S4. Similarity analysis between samples (Heat map).

Figure S5. Heat map of differentially expressed genes (DEGs).

Figure S6. GO significant enrichment analysis for differentially expressed genes (DEGs) during postembryonic development. **a)** B2 vs. O; **b)** B3 vs. B2; **c)** B4 vs. B3; **d)** B5 vs. B4.

Figure S7. KEGG enrichment analysis of DEGs during postembryonic development. The numbers above bars represent the number of enriched genes. **a)** B2 vs. O; **b)** B3 vs. B2; **c)** B4 vs. B3; **d)** B5 vs. B4.

Figure S8. KEGG significant enrichment analysis for differentially expressed genes (DEGs) during postembryonic development. **a)** B2 vs. O; **b)** B3 vs. B2; **c)** B4 vs. B3; **d)** B5 vs. B4.

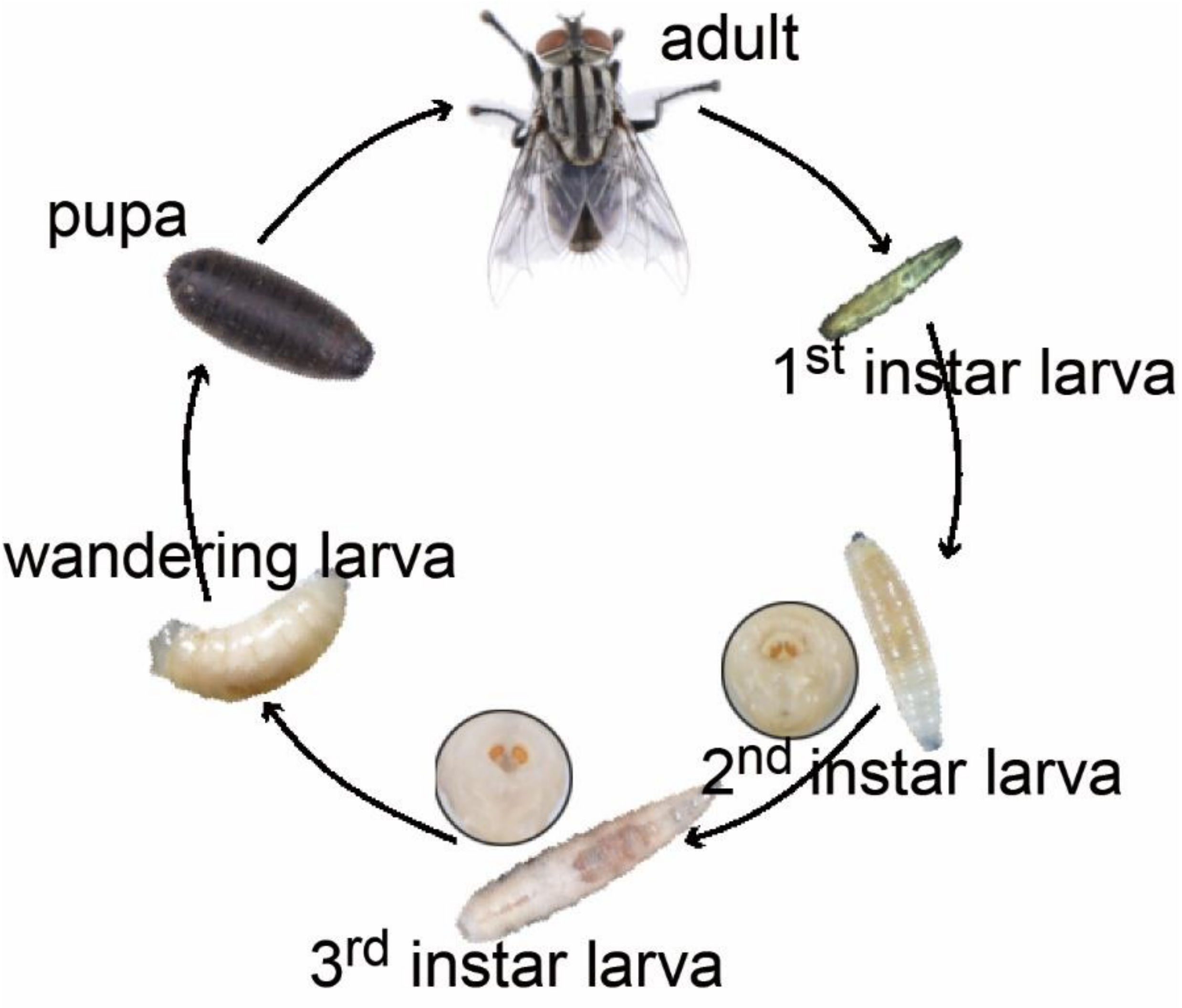

Figure S1. The life cycle of *S. peregrina*.

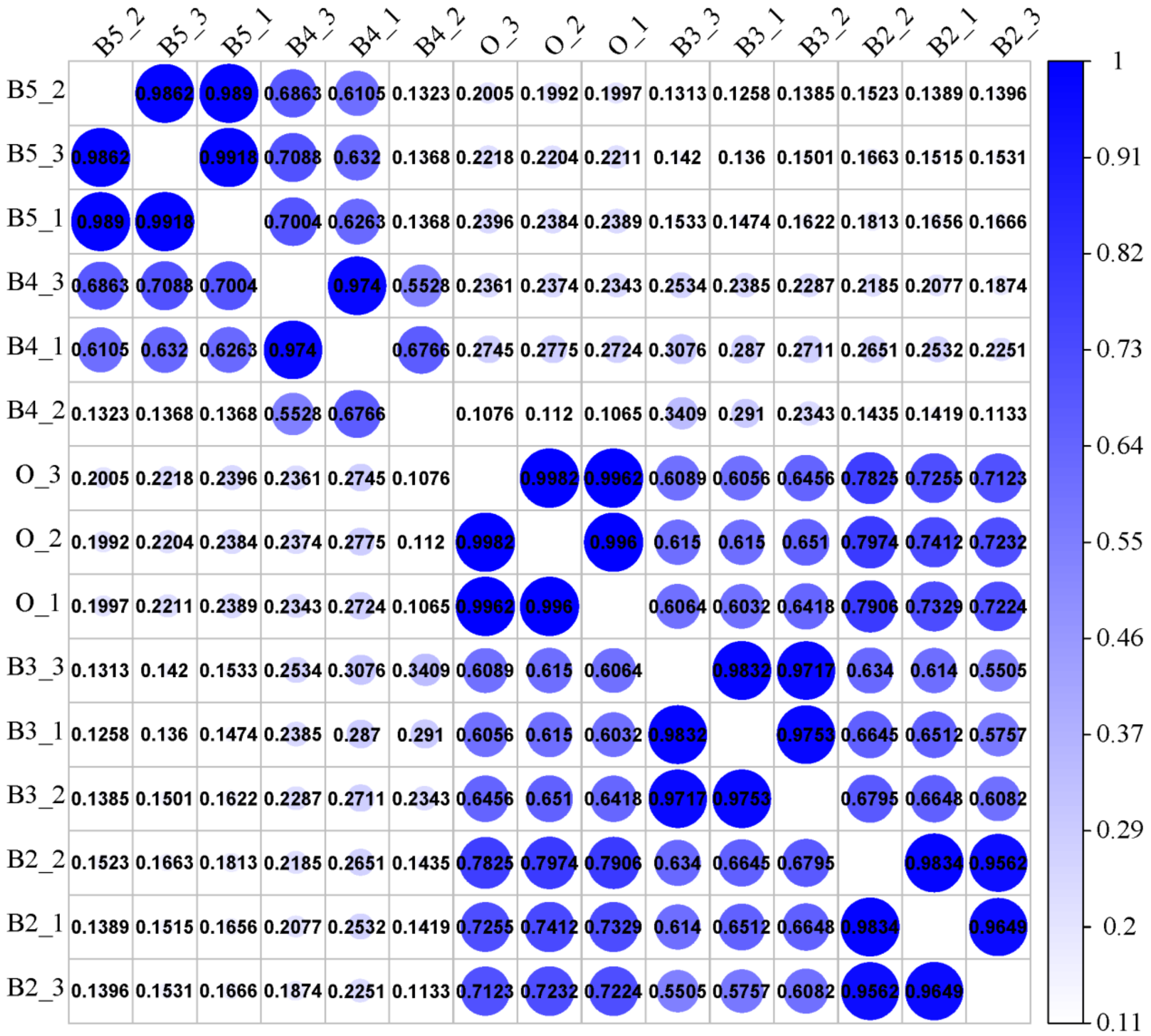

Figure S2. Heat map of correlation analysis between samples. The correlation of biological replicates reflects the degree of similarity between samples. The closer the correlation coefficient is to 1, the higher the similarity between samples is, and the smaller the difference between samples is. X-axis represents the name of the sample, Y-axis represents the name of the corresponding sample, and the scale represents the size of the correlation coefficient.

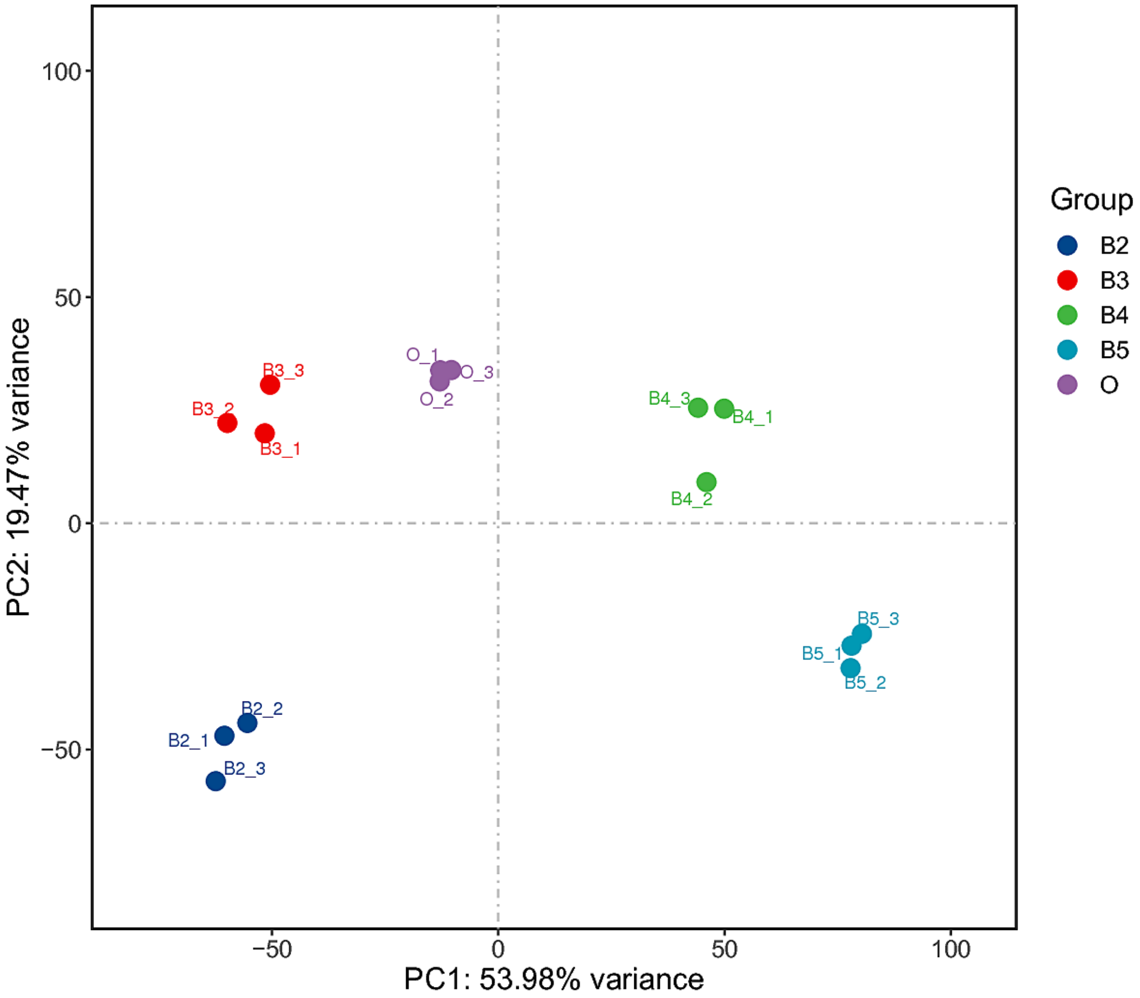

Figure S3. PCA based on the expression level of genes. The closer the PCA variance is, the more similar the samples are.

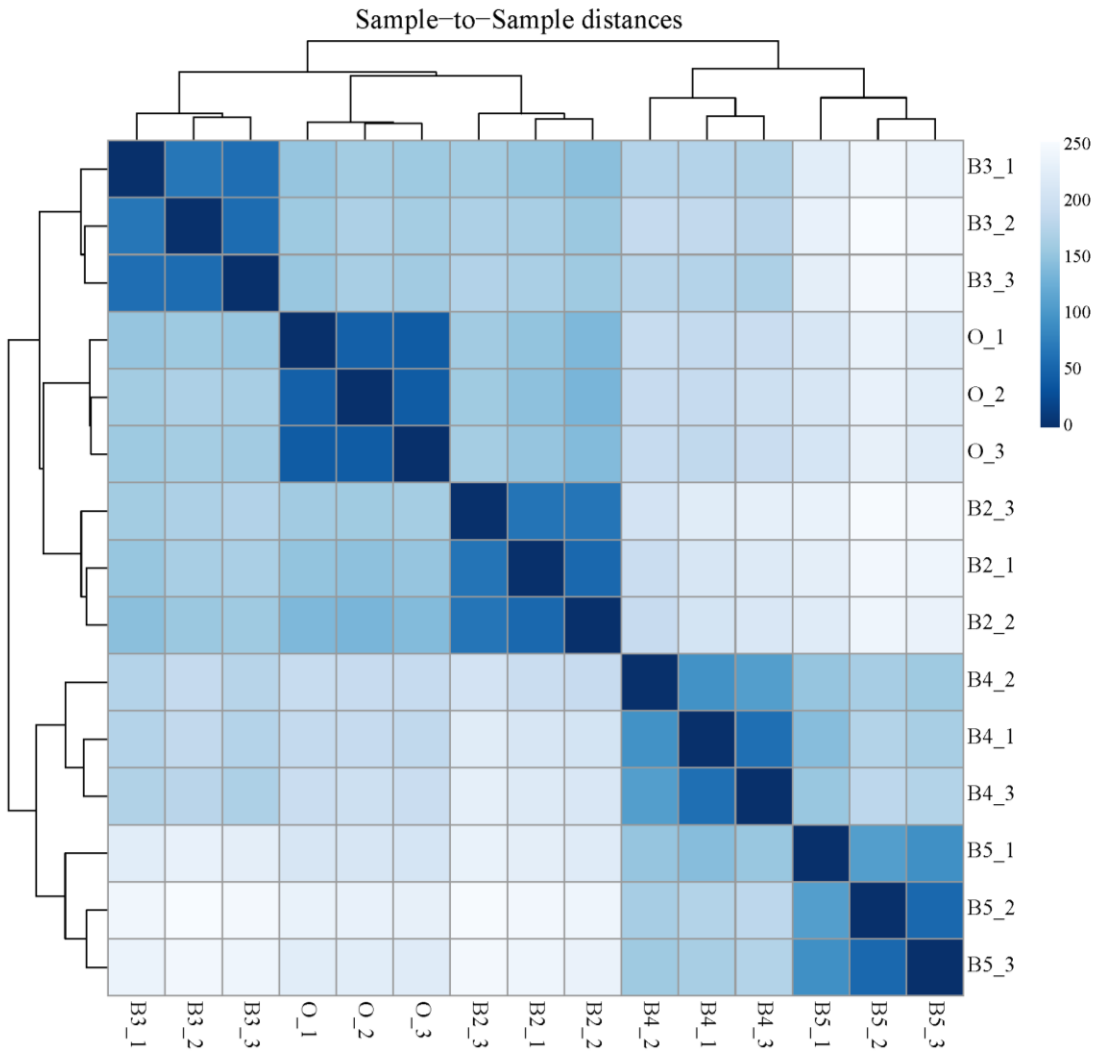

Figure S4. Similarity analysis between samples (Heat map). The analysis can accurately reflect the situation of experimental design, and the distances between samples belonging to the same design are close to each other and preferentially clustered together. X-axis represents the name of the sample, Y-axis represents the name of the corresponding sample, and the scale represents the size of the correlation coefficient.

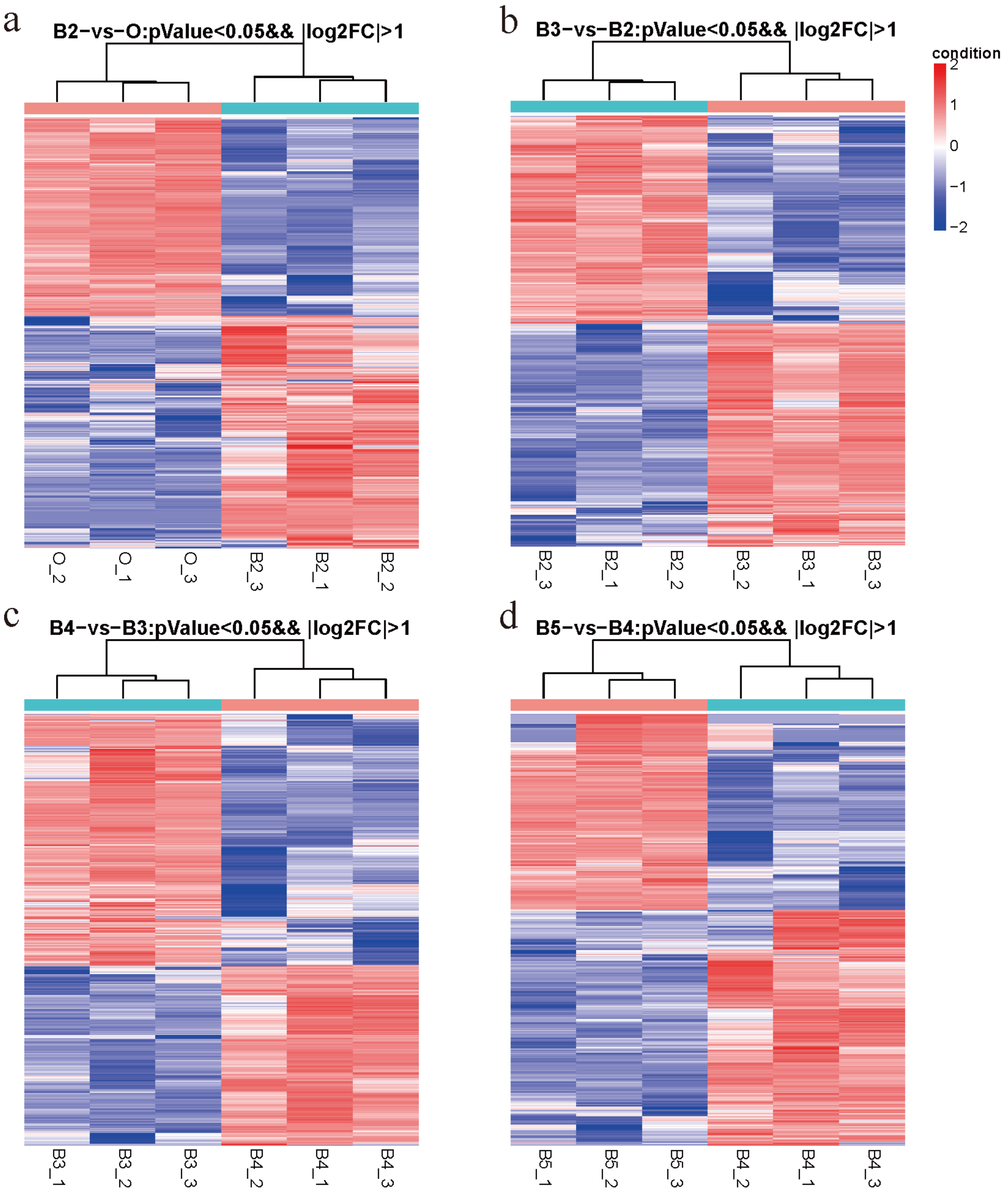

Figure S5. Heat map of differentially expressed genes (DEGs).

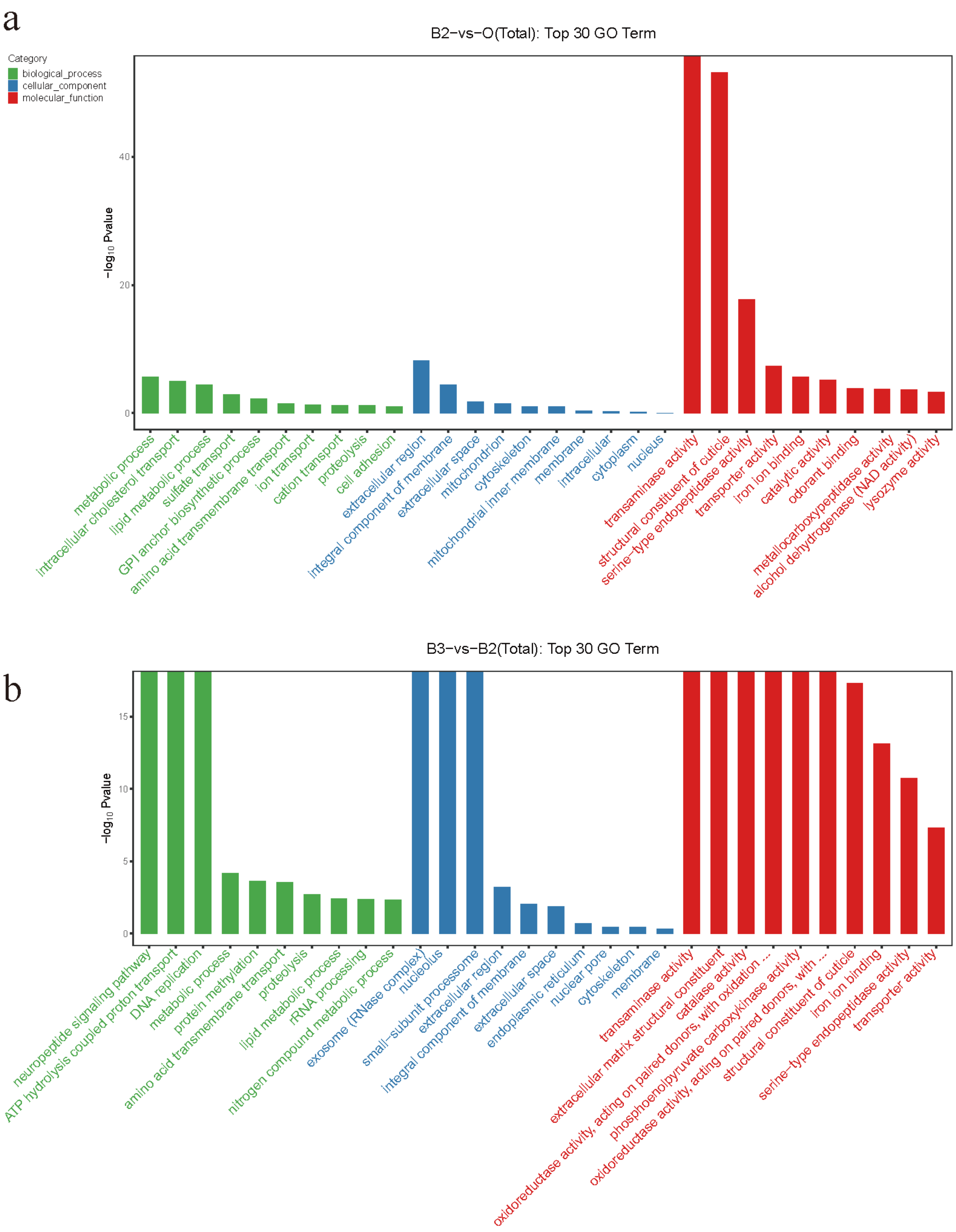
a
B2−vs−O(Total): Top 30 GO Term
Category
biological_process
cellular_component
molecular_function
−log10 Pvalue
0
20
40
metabolic process
intracellular cholesterol transport
lipid metabolic process
sulfate transport
GPI anchor biosynthetic process
amino acid transmembrane transport
ion transport
cation transport
proteolysis
cell adhesion
extracellular region
integral component of membrane
extracellular space
mitochondrion
cytoskeleton
mitochondrial inner membrane
membrane
intracellular
cytoplasm
nucleus
transaminase activity
structural constituent of cuticle
serine−type endopeptidase activity
transporter activity
iron ion binding
catalytic activity
odorant binding
metallocarboxypeptidase activity
alcohol dehydrogenase (NAD activity)
lysozyme activity
b
B3−vs−B2(Total): Top 30 GO Term
−log10 Pvalue
0
5
10
15
neuropeptide signaling pathway
ATP hydrolysis coupled proton transport
DNA replication
metabolic process
protein methylation
amino acid transmembrane transport
proteolysis
lipid metabolic process
rRNA processing
nitrogen compound metabolic process
exosome (RNase complex)
nucleolus
small−subunit processome
extracellular region
integral component of membrane
extracellular space
endoplasmic reticulum
nuclear pore
cytoskeleton
membrane
transaminase activity
extracellular matrix structural constituent
catalase activity
oxidoreductase activity, acting on paired donors, with oxidation ...
phosphoenolpyruvate carboxykinase activity
oxidoreductase activity, acting on paired donors, with ...
structural constituent of cuticle
iron ion binding
serine−type endopeptidase activity
transporter activity

c

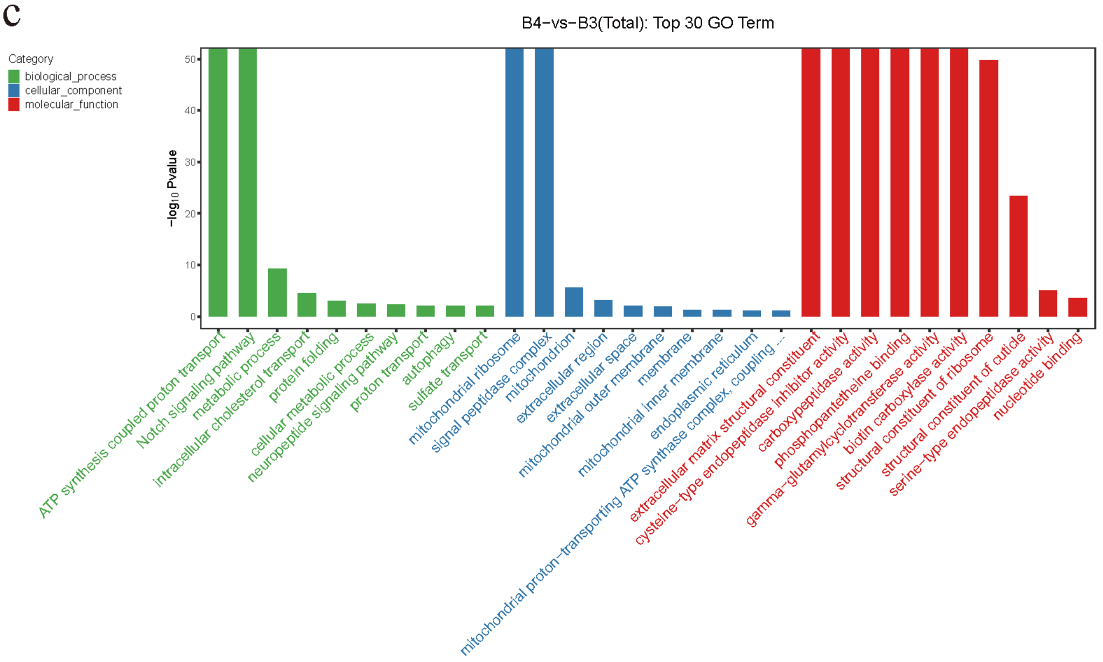

d

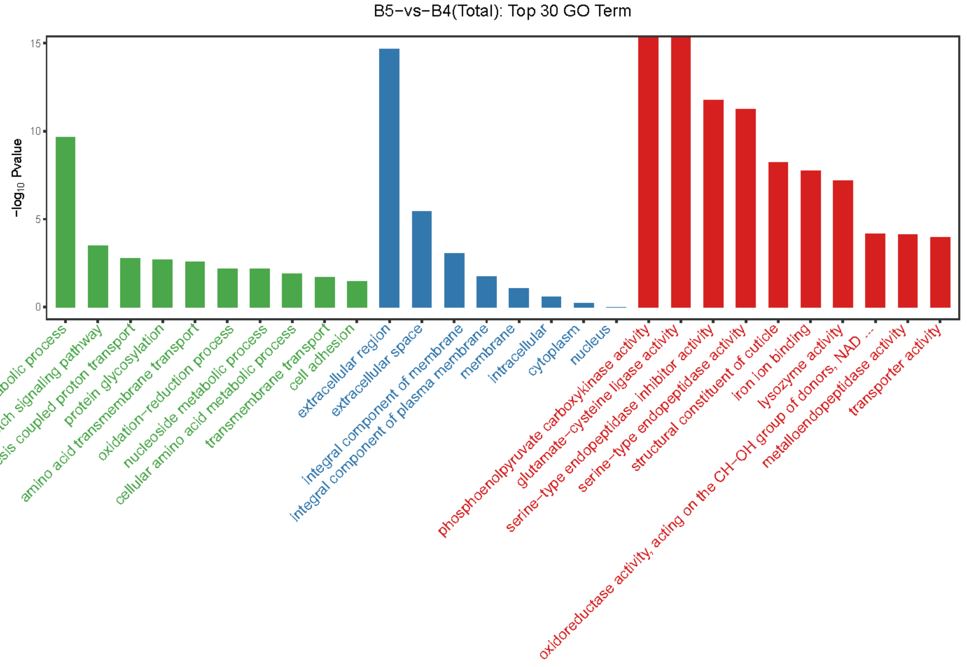

Figure S6. GO significant enrichment analysis for differentially expressed genes (DEGs) during postembryonic development. **a)** B2 vs. O; **b)** B3 vs. B2; **c)** B4 vs. B3; **d)** B5 vs. B4.

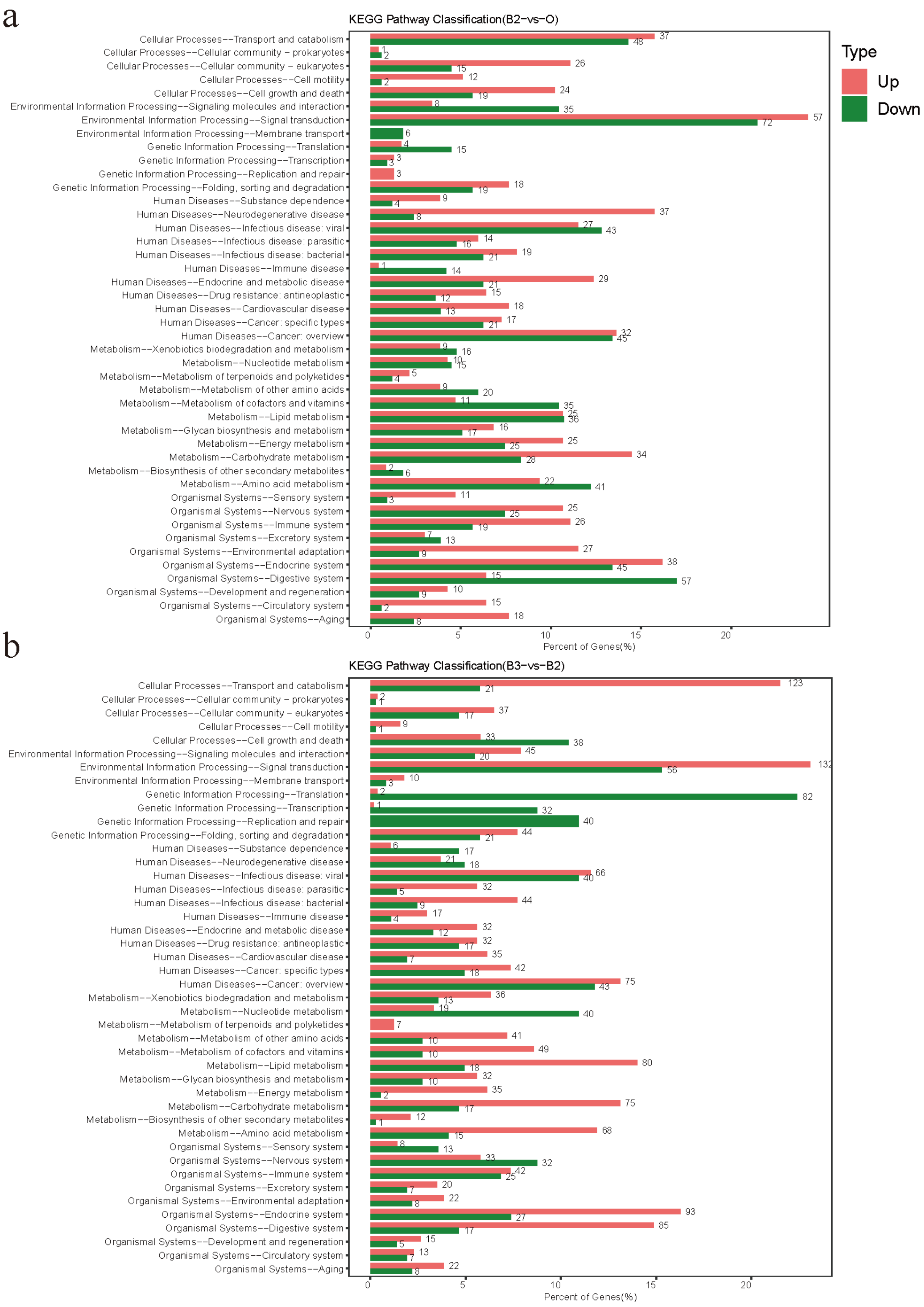
a
KEGG Pathway Classification(B2−vs−O)
Cellular Processes−−Transport and catabolism
Cellular Processes−−Cellular community − prokaryotes
Cellular Processes−−Cellular community − eukaryotes
Cellular Processes−−Cell motility
Cellular Processes−−Cell growth and death
Environmental Information Processing−−Signaling molecules and interaction
Environmental Information Processing−−Signal transduction
Environmental Information Processing−−Membrane transport
Genetic Information Processing−−Translation
Genetic Information Processing−−Transcription
Genetic Information Processing−−Replication and repair
Genetic Information Processing−−Folding, sorting and degradation
Human Diseases−−Substance dependence
Human Diseases−−Neurodegenerative disease
Human Diseases−−Infectious disease: viral
Human Diseases−−Infectious disease: parasitic
Human Diseases−−Infectious disease: bacterial
Human Diseases−−Immune disease
Human Diseases−−Endocrine and metabolic disease
Human Diseases−−Drug resistance: antineoplastic
Human Diseases−−Cardiovascular disease
Human Diseases−−Cancer: specific types
Human Diseases−−Cancer: overview
Metabolism−−Xenobiotics biodegradation and metabolism
Metabolism−−Nucleotide metabolism
Metabolism−−Metabolism of terpenoids and polyketides
Metabolism−−Metabolism of other amino acids
Metabolism−−Metabolism of cofactors and vitamins
Metabolism−−Lipid metabolism
Metabolism−−Glycan biosynthesis and metabolism
Metabolism−−Energy metabolism
Metabolism−−Carbohydrate metabolism
Metabolism−−Biosynthesis of other secondary metabolites
Metabolism−−Amino acid metabolism
Organismal Systems−−Sensory system
Organismal Systems−−Nervous system
Organismal Systems−−Immune system
Organismal Systems−−Excretory system
Organismal Systems−−Environmental adaptation
Organismal Systems−−Endocrine system
Organismal Systems−−Digestive system
Organismal Systems−−Development and regeneration
Organismal Systems−−Circulatory system
Organismal Systems−−Aging
0
5
10
15
20
Percent of Genes(%)
Type
Up
Down
b
KEGG Pathway Classification(B3−vs−B2)
Cellular Processes−−Transport and catabolism
Cellular Processes−−Cellular community − prokaryotes
Cellular Processes−−Cellular community − eukaryotes
Cellular Processes−−Cell motility
Cellular Processes−−Cell growth and death
Environmental Information Processing−−Signaling molecules and interaction
Environmental Information Processing−−Signal transduction
Environmental Information Processing−−Membrane transport
Genetic Information Processing−−Translation
Genetic Information Processing−−Transcription
Genetic Information Processing−−Replication and repair
Genetic Information Processing−−Folding, sorting and degradation
Human Diseases−−Substance dependence
Human Diseases−−Neurodegenerative disease
Human Diseases−−Infectious disease: viral
Human Diseases−−Infectious disease: parasitic
Human Diseases−−Infectious disease: bacterial
Human Diseases−−Immune disease
Human Diseases−−Endocrine and metabolic disease
Human Diseases−−Drug resistance: antineoplastic
Human Diseases−−Cardiovascular disease
Human Diseases−−Cancer: specific types
Human Diseases−−Cancer: overview
Metabolism−−Xenobiotics biodegradation and metabolism
Metabolism−−Nucleotide metabolism
Metabolism−−Metabolism of terpenoids and polyketides
Metabolism−−Metabolism of other amino acids
Metabolism−−Metabolism of cofactors and vitamins
Metabolism−−Lipid metabolism
Metabolism−−Glycan biosynthesis and metabolism
Metabolism−−Energy metabolism
Metabolism−−Carbohydrate metabolism
Metabolism−−Biosynthesis of other secondary metabolites
Metabolism−−Amino acid metabolism
Organismal Systems−−Sensory system
Organismal Systems−−Nervous system
Organismal Systems−−Immune system
Organismal Systems−−Excretory system
Organismal Systems−−Environmental adaptation
Organismal Systems−−Endocrine system
Organismal Systems−−Digestive system
Organismal Systems−−Development and regeneration
Organismal Systems−−Circulatory system
Organismal Systems−−Aging
0
5
10
15
20
Percent of Genes(%)

c

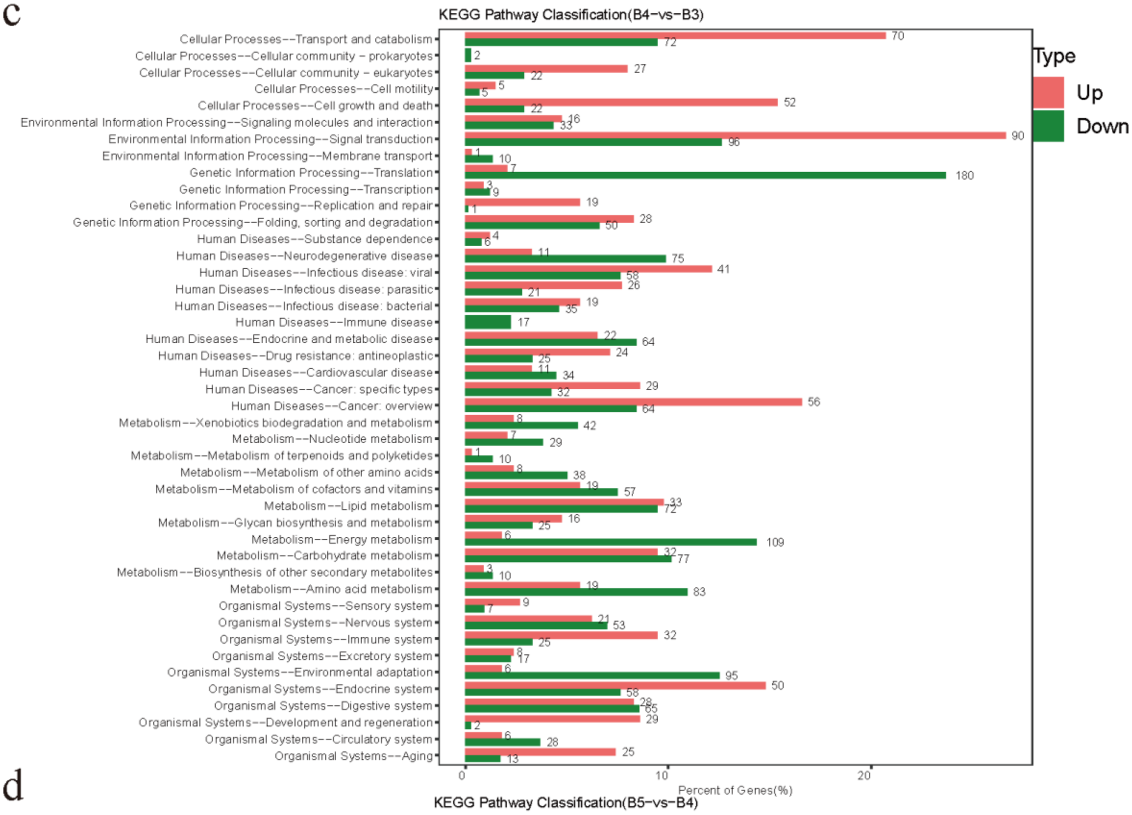

d

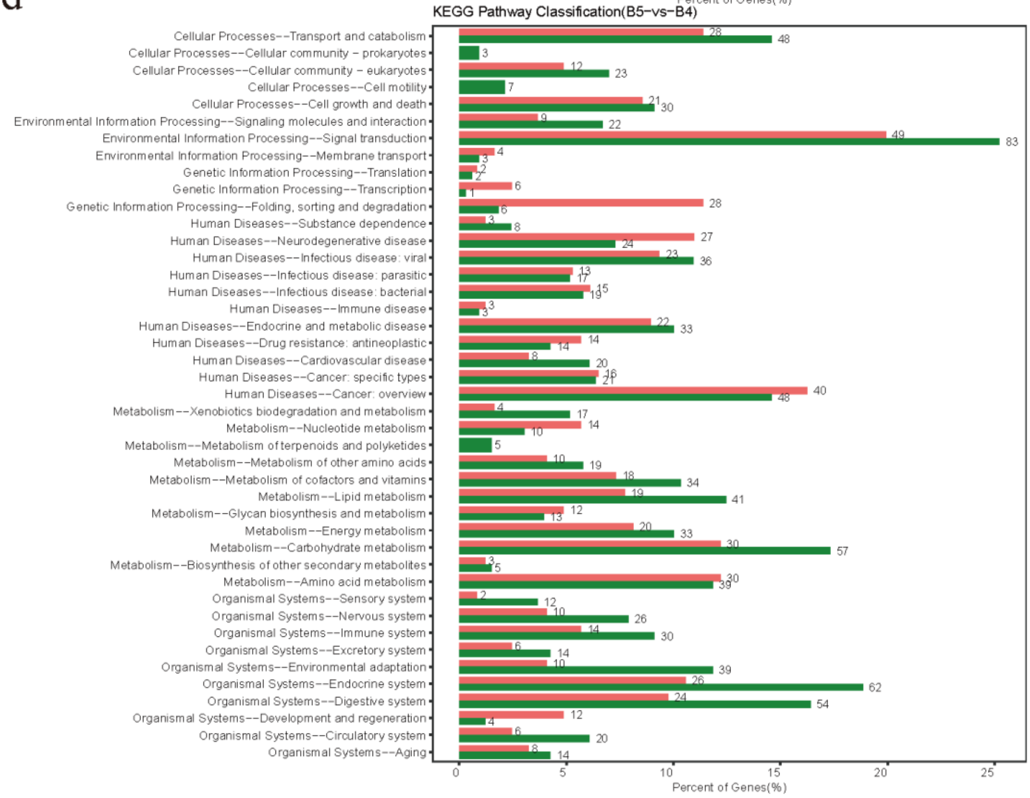

Figure S7. KEGG enrichment analysis of DEGs during postembryonic development. The numbers above bars represent the number of enriched genes. **a)** B2 vs. O; **b)** B3 vs. B2; **c)** B4 vs. B3; **d)** B5 vs. B4.

a

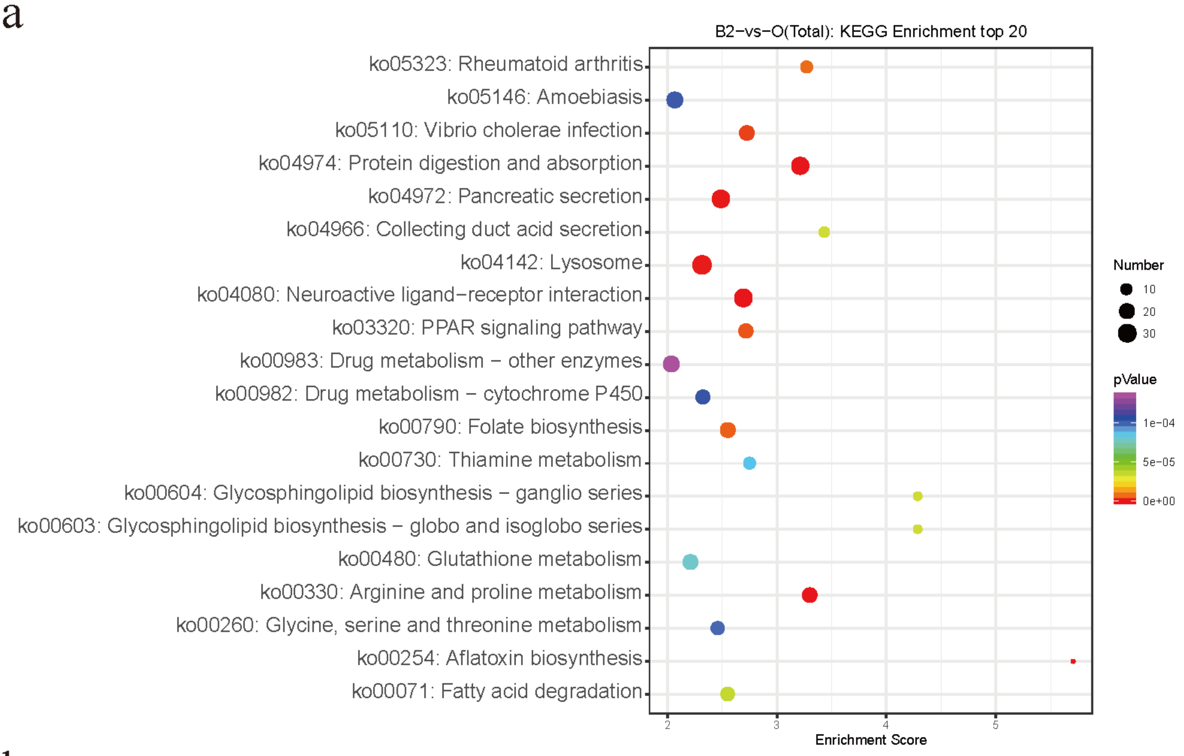
B2−vs−O(Total): KEGG Enrichment top 20
ko05323: Rheumatoid arthritis
ko05146: Amoebiasis
ko05110: Vibrio cholerae infection
ko04974: Protein digestion and absorption
ko04972: Pancreatic secretion
ko04966: Collecting duct acid secretion
ko04142: Lysosome
ko04080: Neuroactive ligand−receptor interaction
ko03320: PPAR signaling pathway
ko00983: Drug metabolism − other enzymes
ko00982: Drug metabolism − cytochrome P450
ko00790: Folate biosynthesis
ko00730: Thiamine metabolism
ko00604: Glycosphingolipid biosynthesis − ganglio series
ko00603: Glycosphingolipid biosynthesis − globo and isoglobo series
ko00480: Glutathione metabolism
ko00330: Arginine and proline metabolism
ko00260: Glycine, serine and threonine metabolism
ko00254: Aflatoxin biosynthesis
ko00071: Fatty acid degradation
2
3
4
5
Enrichment Score
Number
10
20
30
pValue
1e−04
5e−05
0e+00

b

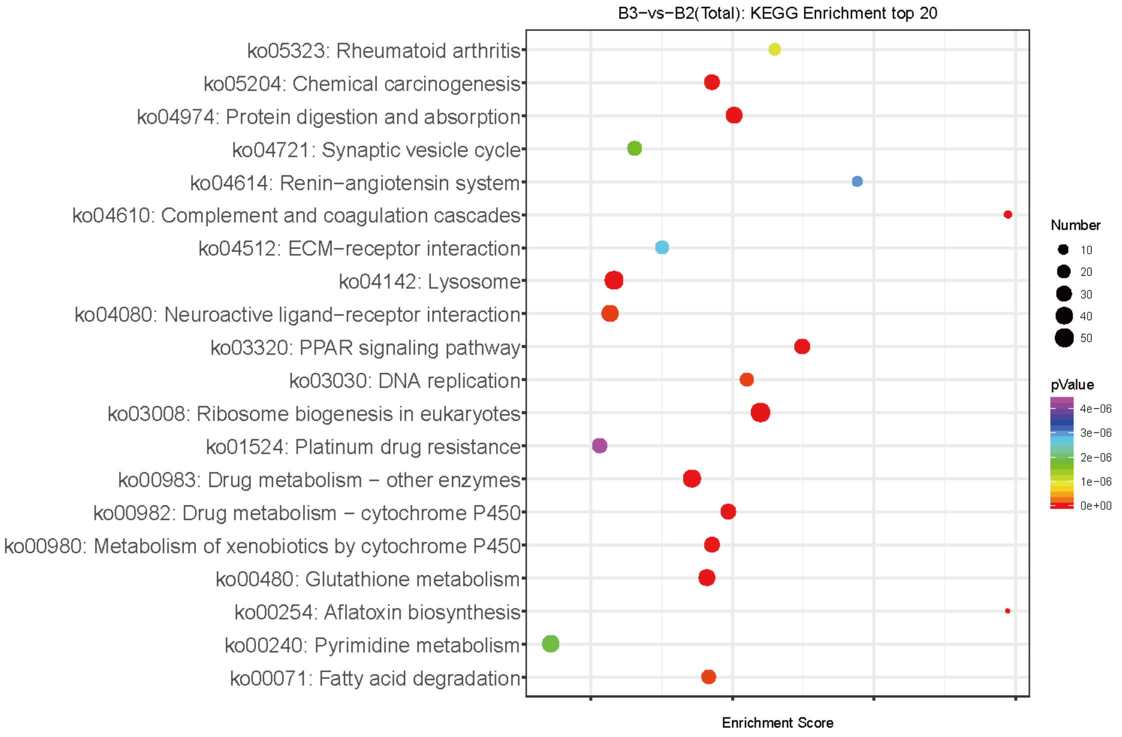
B3−vs−B2(Total): KEGG Enrichment top 20
ko05323: Rheumatoid arthritis
ko05204: Chemical carcinogenesis
ko04974: Protein digestion and absorption
ko04721: Synaptic vesicle cycle
ko04614: Renin−angiotensin system
ko04610: Complement and coagulation cascades
ko04512: ECM−receptor interaction
ko04142: Lysosome
ko04080: Neuroactive ligand−receptor interaction
ko03320: PPAR signaling pathway
ko03030: DNA replication
ko03008: Ribosome biogenesis in eukaryotes
ko01524: Platinum drug resistance
ko00983: Drug metabolism − other enzymes
ko00982: Drug metabolism − cytochrome P450
ko00980: Metabolism of xenobiotics by cytochrome P450
ko00480: Glutathione metabolism
ko00254: Aflatoxin biosynthesis
ko00240: Pyrimidine metabolism
ko00071: Fatty acid degradation
Enrichment Score
Number
10
20
30
40
50
pValue
4e−06
3e−06
2e−06
1e−06
0e+00

c

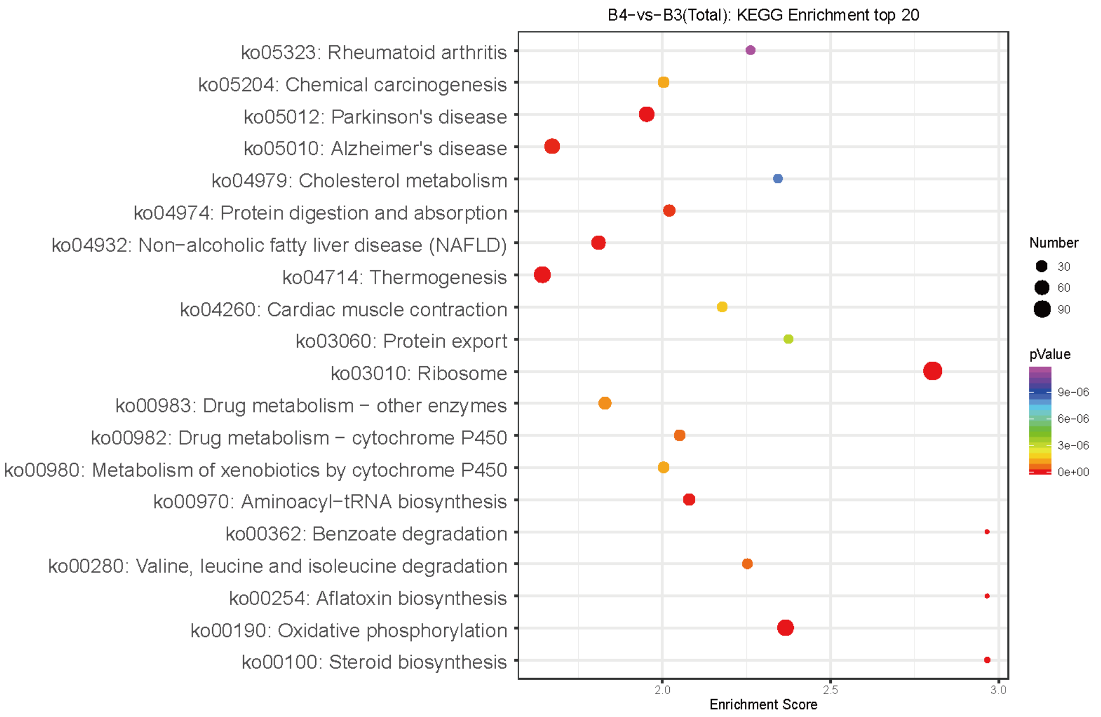

d

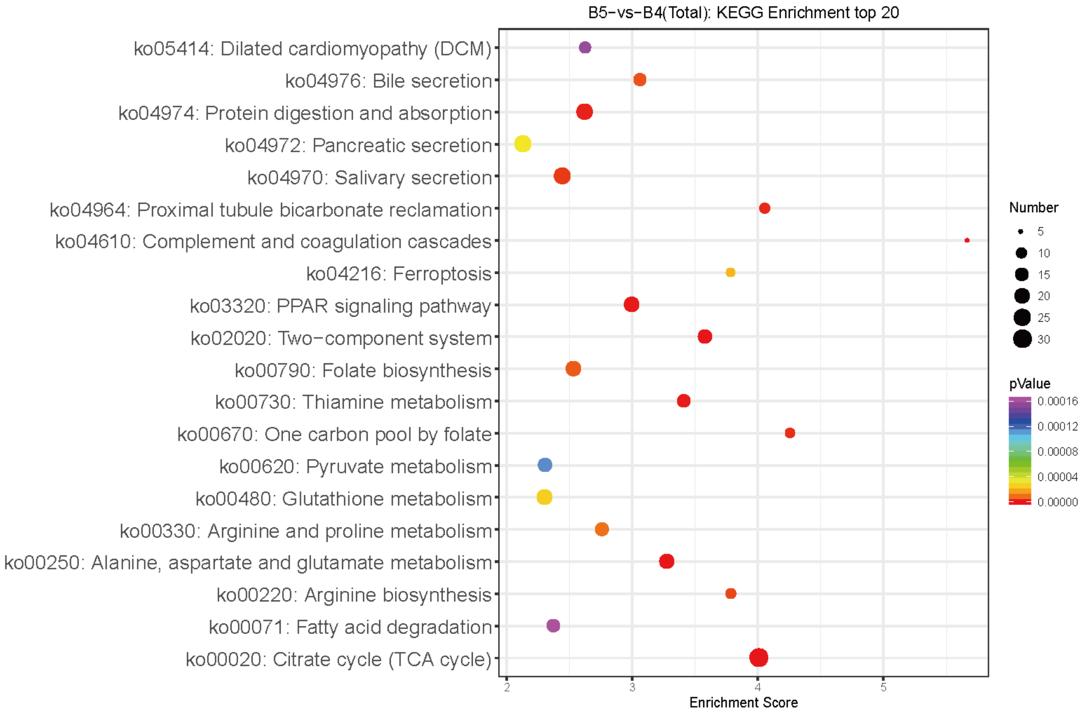

Figure S8. KEGG significant enrichment analysis for differentially expressed genes (DEGs) during postembryonic development. **a)** B2 vs. O; **b)** B3 vs. B2; **c)** B4 vs. B3; **d)** B5 vs. B4.
